# Deciphering the contributions of hybridization and genome dosage to vegetative vigor in perennial forage maize

**DOI:** 10.1111/tpj.71242

**Published:** 2026-11-10

**Authors:** Yingzheng Li, Xu Yan, Zizhou Wu, Qiyuan Zhang, Jiashun Lin, Yong Zhou, Saifei Duan, Yang Zhou, Mingjun Cheng, Huaxiong Li, Xiaofeng Li, Ruyu He, Jianmei He, Xuemei Ni, Huan Liu, Tingzhao Rong, Qilin Tang

**Affiliations:** ^1^ Maize Research Institute Sichuan Agricultural University Chengdu Sichuan 611130 China; ^2^ Crop Research Institute Sichuan Academy of Agricultural Sciences (Germplasm Center of Sichuan Province)/Crop Germplasm Innovation and Genetic Improvement Key Laboratory of Sichuan Province/Environment‐Friendly and Efficient Water‐Saving Technology and Equipment for Hilly Agriculture Key Laboratory of Sichuan Province Chengdu Sichuan 610066 China; ^3^ Institute of Special Economic Animals and Plants Sichuan Academy of Agricultural Sciences/Forage Crops Germplasm Innovation and Production Management Key Laboratory of Nanchong City, Sericulture Research Institute, Sichuan Academy of Agricultural Sciences Nanchong Sichuan 637000 China; ^4^ Sichuan Grassland Technology Research and Promotion Center Chengdu Sichuan 610041 China; ^5^ The People's Government of Dongxing District Neijiang Sichuan 641100 China; ^6^ Agricultural Genomics Institute at Shenzhen Chinese Academy of Agricultural Sciences Shenzhen Guangdong 518000 China; ^7^ Horticulture Research Institute Sichuan Academy of Agricultural Sciences Chengdu Sichuan 610066 China; ^8^ BGI Research Shenzhen Guangdong 518083 China; ^9^ Guangdong Provincial Key Laboratory of Core Collection of Crop Genetic Resources Research and Application Shenzhen Guangdong 518120 China; ^10^ Laboratory of Genomics Ministry of Agriculture Shenzhen Guangdong 518120 China

**Keywords:** allopolyploid, heterosis, genome dosage, transcriptional remodeling, *Zea–Tripsacum*, perennial forage maize

## Abstract

Heterosis and genomic dosage each enhance plant vigor, yet their manifestation and underlying mechanisms in vegetative growth, particularly within polyploid systems, remain unclear. We synthesized and analyzed a series of *Zea*–*Tripsacum* allopolyploid trihybrids (MTPs) with varying genomic compositions to dissect the synergistic contributions of hybridization and polyploidy/aneuploidy effects to vegetative vigor. We demonstrate that increased *Z. perennis* genome dosage directly enhances fresh weight (FW) and photosynthetic capacity, whereas interspecific hybridization‐triggered extensive transcriptome reprogramming. Heterotic hybrids exhibited transcriptional signatures of improved energy use efficiency, characterized by the downregulation of energy‐costly protein quality control systems and upregulation of energy metabolism and carbon fixation pathways. Co‐expression network analysis linked modules of fructose metabolism and glycolysis positively to FW heterosis, while protein folding modules were negatively correlated. Field trials confirmed the agronomic superiority of elite MTP‐derived perennial forage cultivars, Yucao 6 and Yucao 9919, which achieved high yields (127.68–142.61 t ha^−1^) across diverse ecological zones, 156.28–186.25% higher than conventional silage maize. These perennial forage cultivars can be harvested for many years after a single planting, substantially lowering production costs and providing a practical option for utilizing sloping and abandoned lands in southern China. Our findings clarify how hybridization and genomic dosage, via transcriptional remodeling, jointly boost plant vigor, offering both a mechanistic framework and a practical breeding roadmap for integrating heterosis and genomic dosage strategies in crop improvement.

## Significance Statement

Understanding how hybridization and genome dosage contribute to plant vigor remains a challenge. Here, we synthesized developed *Zea–Tripsacum* allopolyploid trihybrids (MTPs) by pyramiding the genomes of *Z. mays*, *T. dactyloides*, and *Z. perennis* to dissect their synergistic effects on vegetative vigor. Our findings elucidate how hybridization and dosage effects synergistically enhance hybrid vigor via transcriptional and metabolic remodeling, providing a roadmap for leveraging genomic dosage strategies in polyploid crop improvement.

## INTRODUCTION

Heterosis and dosage effects are two powerful genetic phenomena utilized in crop breeding. Hybridization confers heterosis, whereas polyploidy generates dosage effects through genome duplication. Integrating these two advantages represents a novel approach to crop improvement.

Heterosis (hybrid vigor) was first described by Darwin (1876), and is widely exploited in maize (Birchler et al., 2010; Crow, 1948; East, 1936; Shull, 1908). It is classified as intraspecific, intersubspecific, or wide‐hybridization heterosis (Fu et al., 2014), with wide‐hybridization showing the greatest potential if issues related to seed set and instability are resolved. Although the use of heterosis has contributed to increased yields in many crops, but the underlying mechanisms remain elusive. Models such as dominance (Bruce, 1910; Jones, 1917; Shen et al., 2014), overdominance (Crow, 1948; East, 1936; Tian et al., 2018), and epistasis (Shen et al., 2014; Torgeman & Zamir, 2023) are supported but incomplete. Omics studies have deepened molecular understanding (Birchler et al., 2010; Hochholdinger & Hoecker, 2007; Liu, He, & Deng, 2021; Okubo & Kaneko, 2022; Wang et al., 2023), yet no consensus exists. Energy use efficiency hypotheses suggest that hybrids reduce protein turnover costs by avoiding misfolded proteins, thereby increasing metabolic efficiency (Bayne, 2004; Ginn, 2010, 2017; Goff, 2011; Goff & Zhang, 2013; Hawkins et al., 1986; Hedgecock et al., 1996), but this remains underexplored in allopolyploids.

Polyploidy offers evolutionary and adaptive advantages (Chen, 2007; Comai, 2005; Leitch & Leitch, 2008; Otto, 2007; Van de Peer et al., 2021). Allopolyploids, as hybrids, exhibit both heterosis and dosage effects (Chen, 2010; June et al., 2023). Important crops such as wheat, cotton, and oilseed rape are allopolyploids, where genome merger and duplication alter gene expression (Chen, 2007, 2010; Doyle et al., 2008). High heterozygosity and intergenomic interactions enhance heterosis (Bingham et al., 1994; Chen, 2007; Li et al., 2014; Ni et al., 2009; Yao et al., 2013), and allopolyploidization can fix heterosis (Chen, 2010). However, classical models struggle to explain polyploid heterosis (Birchler et al., 2010), and dosage effects are often overlooked. For example, maize with an AABB genome exhibits greater heterosis than AAAB or BBBA (Chase, 1980), and heterosis patterns in triploids differ from those in diploids (Yao et al., 2013). Dosage effects also influence phenotype and growth (He et al., 2022; Yu et al., 2024; Zhang et al., 2025), with polyploid advantages including gigantism, enhanced metabolite production, and increased adaptability (Fox et al., 2020; Madani et al., 2021; Orr‐Weaver, 2015; Schoenfelder & Fox, 2015). Nevertheless, distinguishing heterosis from dosage effects remains challenging. Isogenic Arabidopsis F_1_ triploids demonstrate ‘heterosis without hybridity’ (Fort et al., 2016), and dosage studies often focus on gene level expression or evolutionary retention (Bastiaanse et al., 2019, 2021; Bird et al., 2023; He et al., 2022; Yu et al., 2024; Zhang et al., 2025; Zhao et al., 2023). The link between dosage and agronomic traits in synthetic polyploids, as well as the relative breeding value of heterosis versus dosage, remains unclear.

The integration of hybrid vigor with the dosage advantages conferred by engineered polyploid genomes represents a transformative strategy in genetics and crop breeding (Roy et al., 2022; Wang et al., 2024). Previously, we developed a library of trihybrids (MTPs) by hybridizing and shuffling the genomes of three species, *Zea mays*, *Z. perennis*, and *T. dactyloides*. We karyotyped each trihybrid and identified chromosomal structural and phenotypic variations. The differences in phenotypic diversity observed among MTP populations suggest that genomic dosage influences phenotypic manifestation (Li et al., 2024). Here, we dissect heterosis and dosage effects in MTPs at both population and individual levels using agronomic, cellular, and photosynthetic traits, coupled with transcriptomic analyses. We establish a breeding framework that synergizes heterosis and dosage effects, leading to the development of high‐yield perennial forage maize cultivars. Our findings provide insights into the evolutionary dynamics of polyploid chromosomes, genomic dosage regulation, and phenotypic diversification, while advancing breeding theories that integrate hybrid vigor and genomic dosage effects for plant improvement.

## RESULTS

### Vegetative heterosis and genome dosage of *Zea*–*Tripsacum* allopolyploids at population level

In our previous study, an allohexaploid MTP74 (2*n* = 74 = 20Zm + 34Td + 20Zp; Zm, Td, and Zp represent chromosomes from maize, *T. dactyloides*, and *Z. perennis*, respectively) was synthesized by pyramiding chromosomes from *Z. mays* (V182, 2*n* = 4*x* = 40Zm), *T. dactyloides* (TP209, 2*n* = 4*x* = 72Td), and *Z. perennis* (9475, 2*n* = 4*x* = 40Zp) via 2*n*+*n* sexual allopolyploidization (Iqbal et al., 2019; Li et al., 2024; Yan, Cheng, et al., 2020) (Figure 1A). An allooctoploid MTP94 was synthesized by merging an unreduced gamete (2*n*) of MTP74 with a reduced gamete (*n*) of *Z. perennis* (Figure 1B). The pop79 and pop99 populations were synthesized by crossing MTP74 and MTP94, respectively, as the female parents with 9475 as the male parent (Figure 1B). Both populations exhibited extensive chromosomal aneuploidy and differential chromosome dosage caused by a subset of approximately *Z. perennis* subgenome (Li et al., 2024) (Tables S1 and S2). Heterosis analysis indicated that for most traits, MTP94 showed higher values than MTP74, suggesting a weak dosage effect (Table S3), although the differences were not statistically significant. Most trihybrids (72.3% in the pop79 population and 66.7% in the pop99 population) showed significant positive better parent heterosis (BPH) for FW (Figure 1C,D), and some other traits also exhibited varying degrees of heterosis, despite large variation across populations and traits (Figure S1). The pop79 had a mean FW of 8.24 kg·pl^−1^ and 52 individuals (80.0%) showing mid‐parent heterosis (MPH) for FW (Tables S4, S5, S8), while the pop99 had a mean FW of 8.40 kg·pl^−1^, with 43 individuals (79.6%) showing MPH (Tables S6–S8).

**Figure 1:**
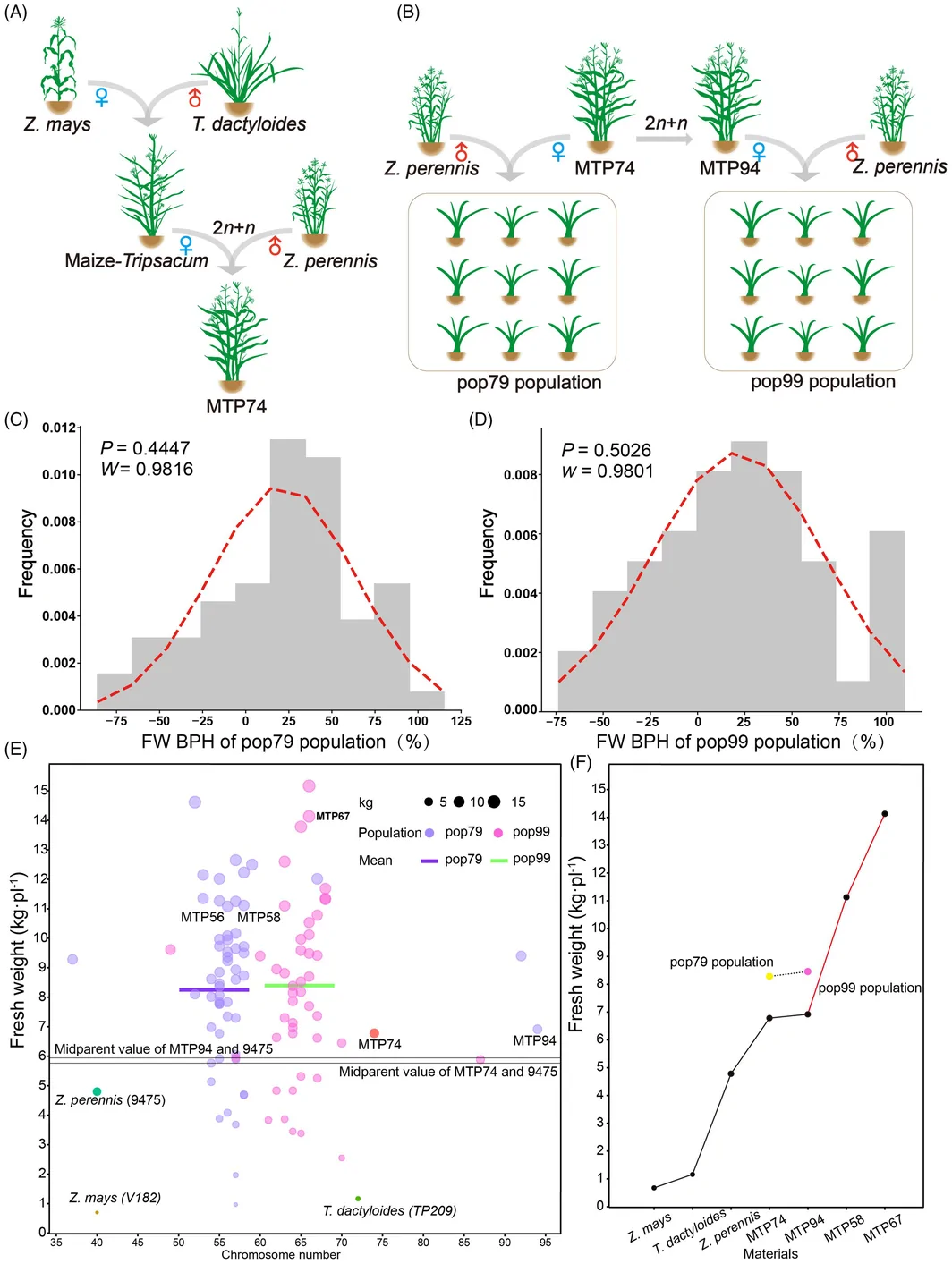
Synthesis roadmap and heterosis in resynthesized pop79 and pop99 allopolyploid populations by chromosome aggregating and shuffling of three species, *Z. mays*, *T. dactyloides*, and *Z. perennis*. (A) Synthesis of the allopolyploid trihybrid MTP74 was achieved by aggregating chromosomes of three species, *Z. mays*, *T. dactyloides*, and *Z. perennis* via 2*n*+*n* sexual allopolyploidization (Iqbal et al., 2019; Yan, Cheng, et al., 2020). (B) MTP94 was synthesized by merging an unreduced gamete (2*n*) of MTP74 with a reduced gamete (*n*) of *Z. perennis*. Furthermore, the pop79 and pop99 populations were synthesized by crossing MTP74 and MTP94, respectively, as female parents with 9475 as the male parent. (C) and (D) Better parent heterosis (BPH) of FW in the pop79 and pop99 populations, respectively. The Shapiro–Wilk test was used to assess normality. (E) Relationship between genome dosage and FW in the synthesized pop79 and pop99 populations. The horizontal axis represents the total chromosome number of each individual. Dot sizes indicate FW magnitude; each dot represents the mean of three independent observations. The purple and green thick lines indicate the mean FW for pop79 and pop99 populations, respectively, while the long lines represent the mid‐parent value of FW in pop99 and pop79 populations. (F) Heterosis observed in different tested plant species and lines generated by aggregating and shuffling the three genomes *Z. mays*, *T. dactyloides*, and *Z. perennis*.

By aggregating the genomes of maize, *T. dactyloides*, and *Z. perennis*, and considering the combined effects of hybridization and polyploidization, MTP74 exhibited FW exceeding any of its three parents (Figure 1E,F). Furthermore, by obtaining two complete *Z. perennis* subgenomes, MTP94 demonstrated increased heterogeneity and ploidy, as well as an increased FW due to the genome dosage effect of *Z. perennis*. The mean FW consistently increased at both the population level (pop99 vs. pop79) and the line level (MTP94 vs. MTP74), further illustrating the genome dosage effect (Figure 1F). This dosage‐dependent enhancement in FW, evident across populations and individual lines, highlights the significant contribution of genomic dosage to vegetative vigor, which operates both independently and synergistically with heterosis derived from hybridization. Therefore, it can be concluded that a high ploidy level and chromosome number play a key role in the heterosis observed in MTPs. Within the pop79 and pop99 populations, chromosomal rearrangements among the three species conferred strong vegetative heterosis to lines such as MTP58 (registered as forage variety Yucao 5), MTP56 (Yucao 6), and MTP67 (Yucao 9919) (Figure 1e).

### Phenotype and karyotype of *Zea*–*Tripsacum* allopolyploids at the representative individual level

Four representative plant materials, MTP56 and MTP57 from the pop79 population, and MTP67 and MTP66 from the pop99 population, were selected for heterosis and karyotype analysis. MTP56 and MTP67 exhibited significant vegetative heterosis, whereas MTP57 and MTP66 displayed hybrid weakness (Figure 2A,B). We examined chromosome compositions based on root‐tip mitosis using multicolor genomic *in situ* hybridization (GISH). In a previous study, we confirmed that the allohexaploid MTP74 has a karyotype of 2*n* = 74 = 20Zm + 34Td + 20Zp (Figure 2C) (Iqbal et al., 2019; Li et al., 2024; Yan, Cheng, et al., 2020), representing an allohexaploid aneuploid missing two *Tripsacum* chromosomes. Thereafter, by mass‐pollinating MTP74 with pollen from *Z. perennis*, we developed the line MTP94, which has a karyotype of 2*n* = 94 = 20Zm + 34Td + 40Zp (Figure 2D). This line was derived via 2*n*+*n* sexual polyploidization, merging an unreduced gamete (2*n*) from MTP74 with a reduced gamete (*n*) from *Z. perennis*. Furthermore, the heterotic lines MTP56 and MTP67 contained 12–17–27 and 11–16–40 chromosomes from *Z. mays*, *T. dactyloides*, and *Z. perennis*, respectively (Figure 2E,F). The hybrid weakness lines MTP57 and MTP66 had 11–16–30 and 10–17–39 chromosomes from *Z. mays*, *T. dactyloides*, and *Z. perennis*, respectively (Figure 2G,H). Clearly, all lines are aneuploid, formed by chromosome shuffling of the two parental lines, MTP74 and MTP94. MTP94 added two complete sets of *Z. perennis* subgenomes. After chromosome shuffling, the major differences between the two superior lines (MTP67 and MTP56) and the two inferior lines (MTP66 vs MTP57) can be attributed to an additional set of *Z. perennis* subgenome, while also considering the influence of other chromosomal rearrangements. However, due to the lack of available chromosome probes for *T. dactyloides* and *Z. perennis*, we were unable to accurately detect which chromosomes were involved in the observed structural and numerical changes. Hybridity and polyploidy resulted in varying subgenome dosages among the tested lines, providing an opportunity to investigate heterosis and genome dosage effects.

**Figure 2:**
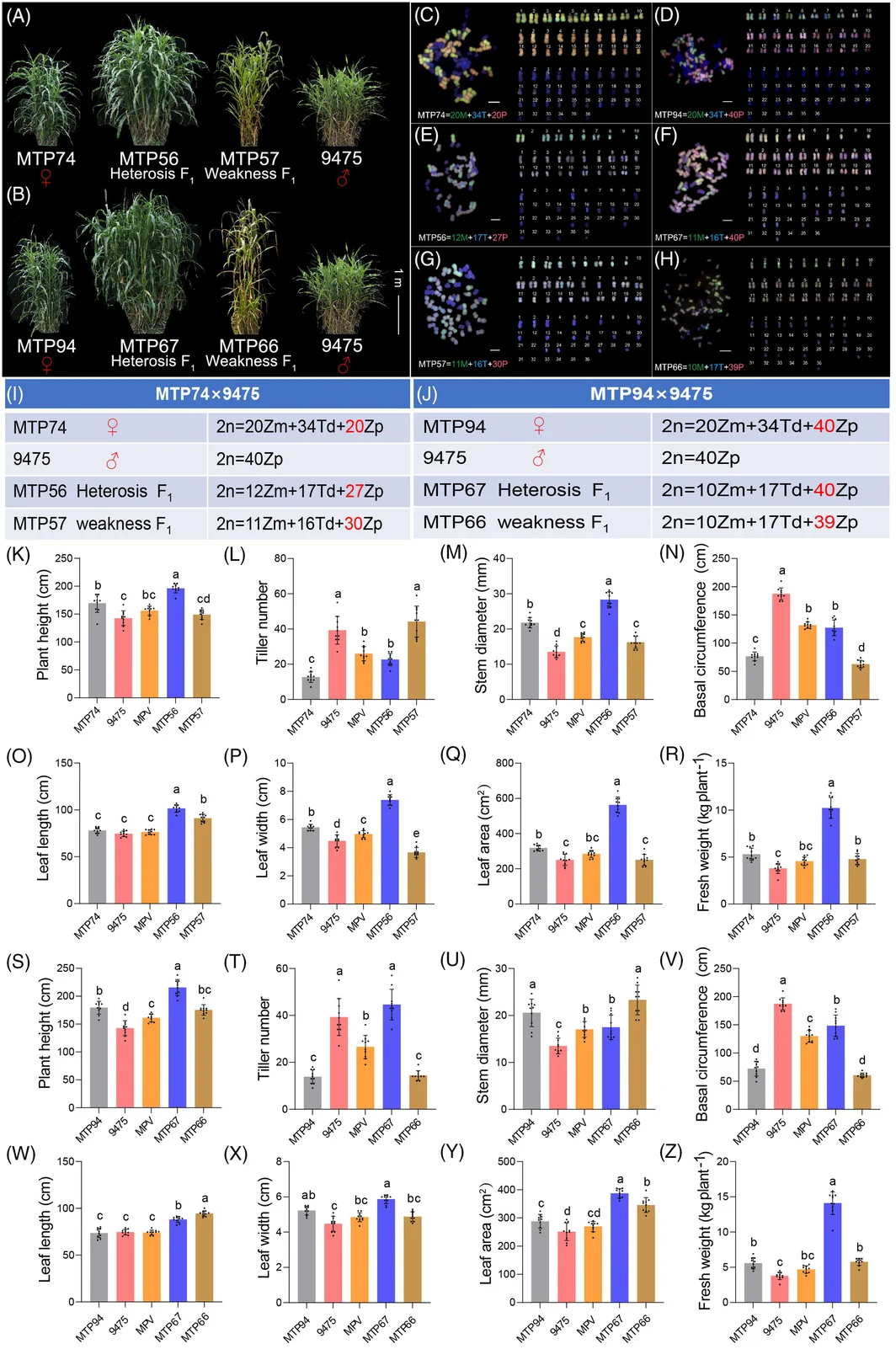
Phenotypes and karyotypes of representative allopolyploid trihybrid F_1_ individuals and their parents from the pop79 and pop99 populations. (A and B) Plant morphology of heterotic and weakness lines selected from pop79 and pop99, along with their parents. (C–H) Mitotic karyotypes of lines MTP74, MTP94, MTP56, MTP67, MTP57, and MTP66. Green, red, and blue colors represent chromosomes of *Z. mays* (Zm), *Z. perennis* (Zp), and *T. dactyloides* (Td), respectively. Scale bar is 10 μm. (I and J) show the karyotype statistics for two heterosis lines, two weakness lines, and their corresponding parents. (K–R) Eight traits observed in the MTP74 × 9475 cross combination. (S–Z) Eight traits observed in the MTP94 × 9475 cross combination. Mid‐parent value (MPV) represents the average trait value of the parents. Single factor ANOVA was used to analyze the significance of differences, and the Turkey method was applied for multiple comparisons. The error bars in the figure represent the standard deviation (SD), the same below. Sample size was 10.

### Vegetative growth heterosis in *Zea*–*Tripsacum* trihybrids

Five allopolyploid trihybrids, namely MTP94 (derived from MTP74 and 9475 with 2*n* +*n*), MTP56 and MTP57 (derived from MTP74 and 9475 with *n* +*n*), and MTP67 and MTP66 (derived from MTP94 and 9475 with *n* +*n*), along with their polyploid parental lines, were used to investigate possible mechanisms of heterosis and dosage effects. *Zea–Tripsacum* trihybrids exhibited phenotypes influenced by their three original parents. For example, the trihybrids are perennial with multiple tillers, similar to *Z. perennis* and *T. dactyloides*, but each tiller is erect and stout like maize (Figure 2A,B). Notably, strong FW heterosis was observed at vegetative stages (105 days after sowing) in MTP56 and MTP67 trihybrids (Figure 2A,B). We compared the FW‐related heterosis for the tested traits using F_1_ hybrids, their parental lines, and the MPV. Among the eight traits evaluated in the MTP74 × 9475 hybrid, MTP56 showed heterosis in plant height, stem diameter, leaf length, leaf width, leaf area, and FW, while MTP57 exhibited heterosis only in tiller number and leaf length; the remaining traits, basal circumference and leaf width, showed hybrid weakness (Figure 2I–P). In the MTP94 × 9475 hybrid, MTP67 displayed heterosis for most traits, including plant height, tiller number, basal circumference, leaf width, leaf area, and FW, whereas MTP66 showed hybrid weakness for all traits except stem diameter, leaf length, and leaf area (Figure 2Q–X). We observed that in two cross combinations, tested lines exhibiting strong heterosis for agronomic traits and yield did not always display heterosis for reproductive traits. For example, among the six reproductive traits measured in the MTP74 × 9475 hybrid, MTP56 showed heterosis only in silk length and silk diameter. In contrast, MTP57 did not exhibit significant hybrid weakness in reproduction‐related traits (Figure S2a–f). A similar pattern was observed in the MTP94 × 9475 hybrid (Figure S2g–l).

Heterosis can likely be attributed to increased cell size, cell number, or both. To investigate this, we measured stomatal size, density, pollen size, chlorophyll content, and photosynthetic capacity in trihybrids and their parental lines (Figures S3 and S4). Among the six cell size traits observed in the MTP74 × 9475 hybrid, MTP56 exhibited heterosis in stomatal width, stomatal area, and stomatal density, while MTP57 showed heterosis only in pollen size; the remaining traits displayed hybrid weakness (Figure S3a–f). In the MTP94 × 9475 hybrid, MTP67 demonstrated slight heterosis in stomatal length, perimeter of guard cells, and stomatal density, whereas MTP66 showed hybrid weakness in stomatal length, perimeter of guard cells, and stomatal density (Figure S3g–l). Moreover, in the MTP74 × 9475 hybrid, MTP56 exhibited significantly higher heterosis than in the MPV for net photosynthetic rate, stomatal conductance, transpiration rate, and intercellular CO_2_ concentration, whereas MTP57 did not show significant heterosis for these parameters (Figure S4a–f). MTP67 showed remarkable heterosis in photosynthetic parameters (Figure S4i–p). In several cases, significant correlations were observed between FW and other traits (Figure S5). A strong positive correlation was observed between FW and plant height (*r* = 0.92), basal circumference (*r* = 0.90), and net photosynthetic rate (*r* = 0.91). Plant height was also significantly positively correlated with basal circumference (*r* = 0.90), suggesting that a more spacious environment promotes growth. As an important feed‐related trait, tiller number and stem diameter exhibited a negative correlation (*r* = −0.63), indicating developmental competition among individual tillers.

### Effects of increased *Z. perennis* genomic dosage on plant phenotype

MTP74 exhibited significantly increased biomass compared to the three parent lines (Figure 1F), and MTP94 contained two complete sets of *Z. perennis* genomes derived from MTP74 (Figure 2D). Similarly, the heterotic F_1_ hybrid (MTP56 vs. MTP67) and the hybrid weakness F_1_ (MTP57 vs. MTP66) showed major differences attributable to the addition of *Z. perennis* genomes (Figure 2E–H). To clarify the extent of the dosage effect of the *Z. perennis* genomes, we further analyzed the three dosage comparison groups (MTP74 vs. MTP94, MTP56 vs. MTP67, and MTP57 vs. MTP66). The increased genome dosage of the *Z. perennis* in MTP94, MTP67, and MTP66 generally resulted in enhanced agronomic traits; however, the magnitude and direction of the dosage effects varied across groups and traits. For instance, the three dosage comparisons indicated that increased genome dosage led to an increase in plant height ranging from 5.90 to 17.64% (Figure 3A). MTP94 showed an 8.66% increase in tiller number compared to MTP74, MTP67 increased by 97.35% compared to MTP56, whereas MTP66 decreased by 67.42% compared to MTP57 (Figure 3B). Regarding stem diameter, MTP94 decreased by 5.65% relative to MTP74, MTP67 decreased by 38.18% relative to MTP56, while MTP66 increased by 43.82% compared to MTP57 (Figure 3C). For basal circumference, MTP94 decreased by 5.48% compared to MTP74, MTP67 increased by 16.65% compared to MTP56, and MTP66 decreased by 3.50% compared to MTP57 (Figure 3D). In terms of leaf length, leaf width, and leaf area, two comparison groups (MTP94 vs. MTP74 and MTP67 vs. MTP56) exhibited a negative dosage effect, whereas MTP66 vs. MTP57 showed a positive dosage effect (Figure 3E–G). The increase in *Z. perennis* genome dosage resulted in a FW increase ranging from 5.08 to 37.99% across the three dosage comparison groups. Furthermore, increased genome dosage of *Z. perennis* generally enhanced most reproduction‐related traits, except for spike length in MTP67 vs. MTP56, and tassel length and tassel branch number in MTP94 vs. MTP74 (Figure S6). Additionally, cell size parameters, including stomatal length, stomatal width, perimeter of guard cells, stomatal area, and pollen size, also increased with higher *Z. perennis* genome dosage, except for stomatal width in MTP67 vs. MTP56 and pollen size in MTP66 vs. MTP57 (Figure S7). However, the stomatal density was negatively correlated with genome dosage in all groups (Figure S7e). Moreover, increased *Z. perennis* genome dosage was associated with a general enhancement in photosynthetic capacity and chlorophyll content (Figure S8).

**Figure 3:**
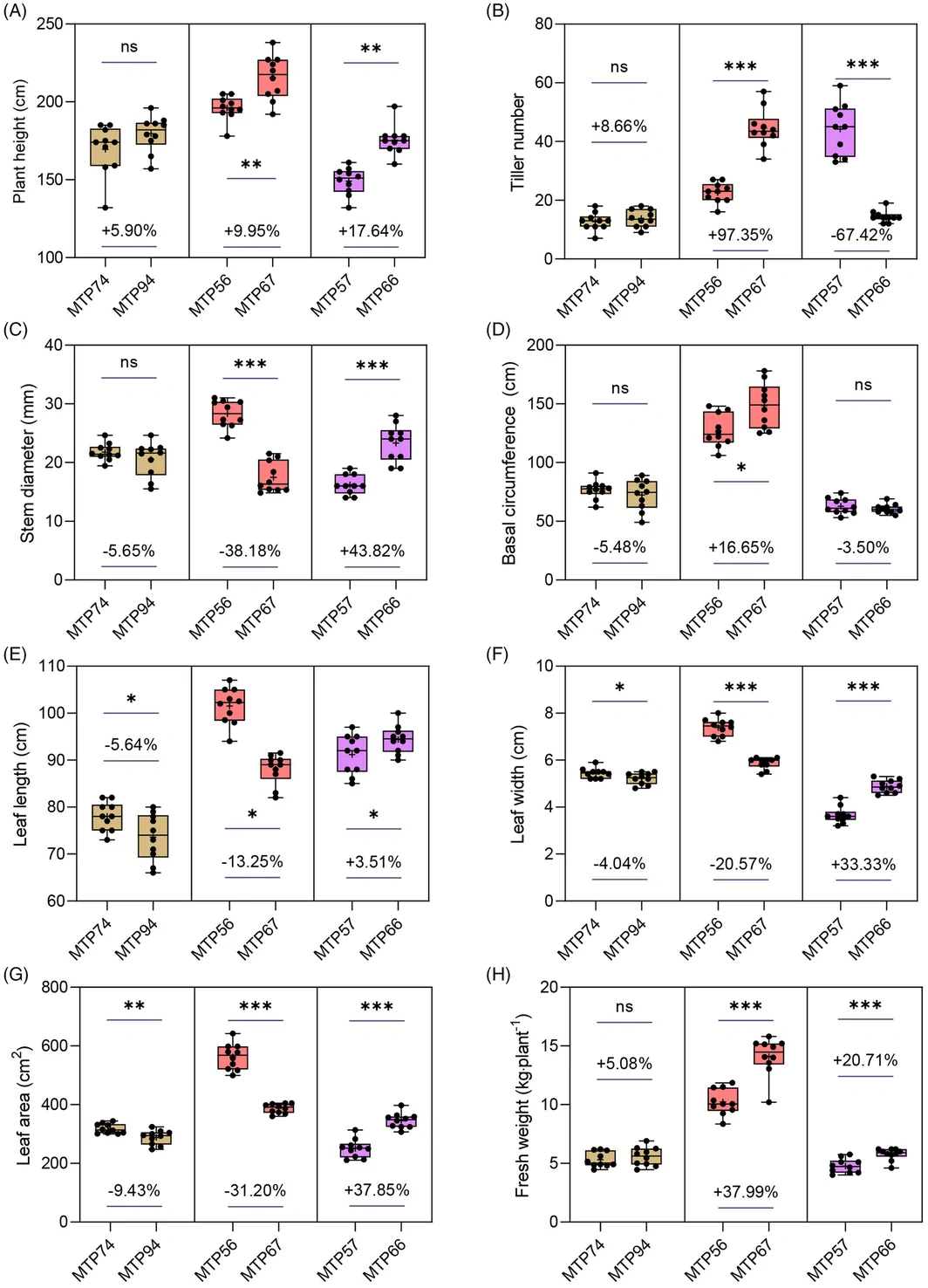
Dosage effect analysis of agronomic traits and yield in representative plant materials from two populations. (A–H) represent plant height, tiller number, stem diameter, basal circumference, leaf length, leaf width, leaf area, and fresh weight. In each dosage comparison, the significance of differences was analyzed using unpaired *t*‐tests, and *P*‐values were calculated using two‐tailed tests. Symbols *, **, ***, and ns denote *P* < 0.05, *P* < 0.01, *P* < 0.001, and *P* > 0.05, respectively. Percentages on the horizontal line indicate the magnitude of the dosage effect on the trait. Sample size: *N* = 10.

### Chromosomal shuffling altered the global transcriptional profile

To investigate how hybridization and genome dosage influence the transcriptomic landscape, we performed RNA sequencing (RNA‐seq). Each sample generated over 10 Gb of sequencing data, with an average of 12.86 Gb per sample. The data quality was high, with an effective data rate exceeding 98.6% and Q20 scores above 97.1%. The average guanine‐cytosine (GC) content across all samples was 55.68%. On average, more than 86.65% of valid reads mapped to the MTP74 reference transcriptome (Table S9). Based on mapping and quantification results, gene expression level (FPKM) was categorized into six groups: 0–0.1 as extremely low expression, 0.1–1 as low expression, 1–5 as moderate expression, 5–15 as high expression, 15–60 as higher expression, and >60 as ultra‐high expression genes. *Z. perennis* exhibited the highest percentages of extremely low expressed genes (44.5%), higher expressed genes (3.1%), and ultra‐high expressed genes (1.0%), while having the lowest percentage of low expressed genes is 26.0%. The allopolyploid trihybrid F_1_ showed an increased proportion of extremely low expressed genes and decreased proportions of low expressed genes and ultra‐high expressed genes compared to MTP74 and MTP94 (Figure S9; Table S10). These results suggest that hybridization and recombination have caused substantial differences in the genetic composition between the allopolyploid trihybrid and its two parents, likely reshaping the global expression landscape. Inter‐sample correlation analysis revealed high correlations ranging from 0.85 to 0.99 (Figure S10).

### Differentially expression genes (DEGs) and their functions

Based on comparative quantification across samples, genes with FPKM values greater than 0.1 in at least one sample of each line were selected for further DEGs analysis (Figure 4A). In the two heterosis combinations (MTP74 × 9475 → MTP56, MTP94 × 9475 → MTP67), the MTP74 vs. MTP56 (control vs. treatment group, hereinafter) comparison revealed 18 436 DEGs, of which 6017 (32.6%) were upregulated and 12 419 (67.4%) were downregulated. The 9475 vs. MTP56 comparison revealed 49 063 DEGs, with 34 129 (69.6%) upregulated and 14 934 (30.4%) downregulated. The MTP94 vs. MTP67 comparison identified 29 068 DEGs, including 10 834 (37.3%) upregulated and 18 234 (62.7%) downregulated; the 9475 vs. MTP67 comparison revealed 53 135 DEGs, of which 35 016 (65.9%) were upregulated and 18 119 (34.1%) were downregulated (Figure 4A). These results indicate that in the two heterosis lines, more DEGs were downregulated compared to their female parent, while more DEGs were upregulated compared to the male parent. In the two hybrid weakness combinations (MTP74 × 9475 → MTP57, MTP94 × 9475 → MTP66), 65 333 DEGs were detected in the MTP74 vs. MTP57 comparison, including 30 541 (46.8%) upregulated and 34 792 (53.2%) downregulated. A total of 55 507 DEGs were identified in 9475 vs. MTP57, with 36 146 (65.1%) upregulated and 19 361 (34.9%) downregulated. The MTP94 vs. MTP66 comparison identified 41 437 DEGs, of which 20 283 (49.0%) were upregulated and 21 154 (51.0%) were downregulated; the 9475 vs. MTP66 comparison revealed 57 725 DEGs, including 37 704 (65.3%) upregulated and 20 021 (34.7%) downregulated. Notably, comparisons involving hybrid weakness lines revealed more DEGs than those involving heterosis materials (Figure 4A). Furthermore, 4764 and 7902 DEGs overlapped in the two heterosis combinations (Figure 4B,C), with 1622 core DEGs shared by both heterosis combinations (Figure 4D). These core DEGs represent potential gene groups associated with heterosis in allopolyploid trihybrids and warrant further investigation. Additionally, 12 628 and 10 953 DEGs overlapped in the two hybrid weakness combinations (Figure 4E,F), with 2940 core DEGs shared by both hybrid weakness combinations (Figure 4G).

**Figure 4:**
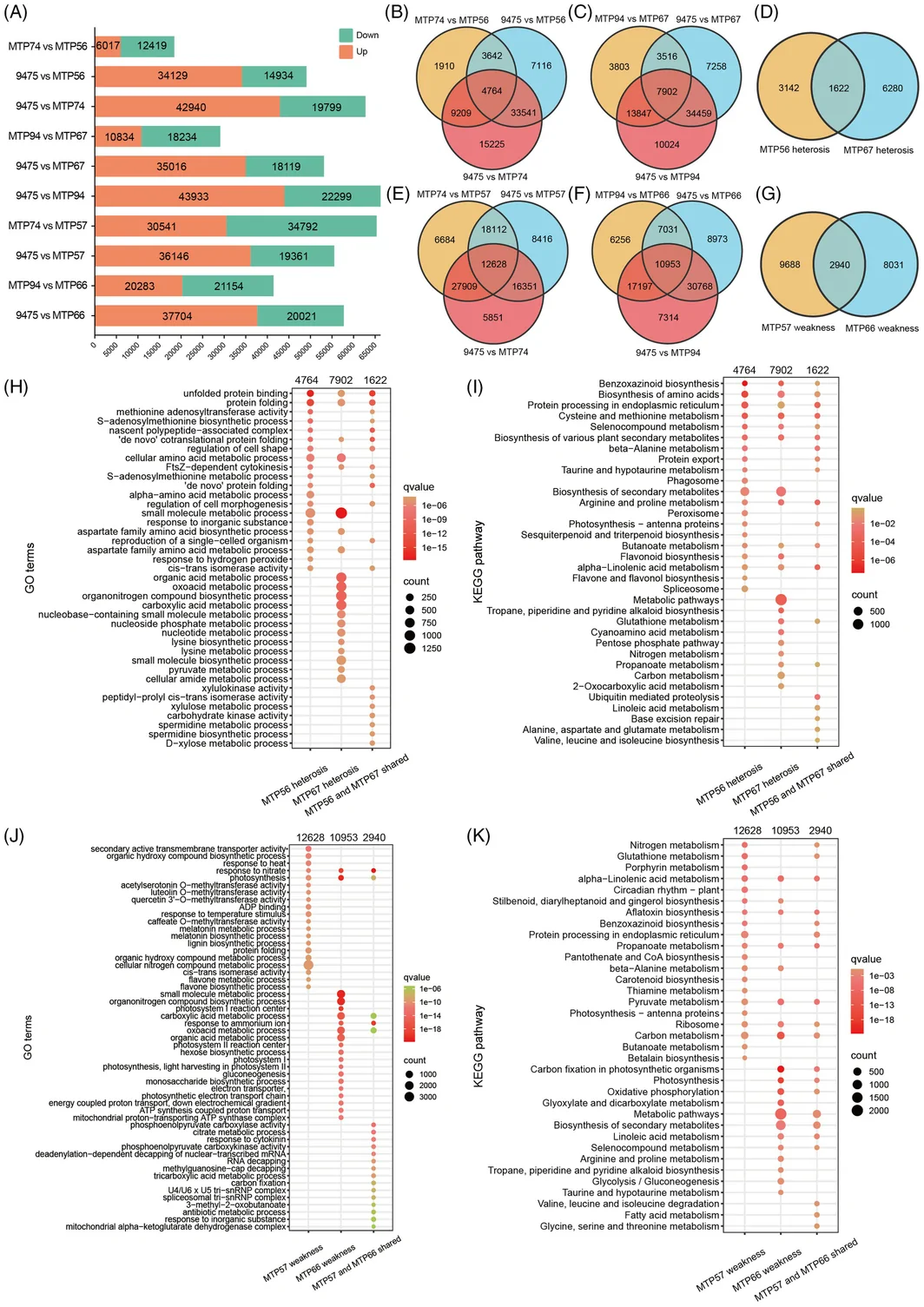
DEG analysis and functional annotation related to heterosis and hybrid weakness in perennial forage maize. (A) Overview of DEGs between the heterosis and hybrid weakness materials and both parents. (B) Venn diagram illustrating the core DEGs in the heterosis combination MTP74 × 9475 → MTP56. (C) Venn diagram illustrating the core DEGs in the heterosis combination MTP94 × 9475 → MTP67. (D) Venn diagram showing the core group of genes shared by the two heterosis combinations. (E) Venn diagram illustrating the core DEGs in the hybrid weakness combination MTP74 × 9475 → MTP57. (F) Venn diagram representing the core DEGs in the hybrid weakness combination MTP94 × 9475 → MTP66. (G) Venn diagram representing the core group of genes shared by the two hybrid weakness combinations. (H and I) represent the top 20 GO functional terms and KEGG pathway annotations, respectively, for the heterosis gene group. (J and K) represent the top 20 GO functional terms and KEGG pathway annotations, respectively, for the hybrid weakness gene group.

To functionally characterize the mechanisms underlying heterosis and hybrid weakness, overlapping and core DEGs were pooled to perform Gene Ontology (GO) and Kyoto Encyclopedia of Genes and Genomes (KEGG) enrichment analyses. In the heterosis combination MTP74 × 9475 → MTP56, 4764 overlapping DEGs were significantly enriched in 168 GO terms (FDR <0.05), with the majority of genes functionally annotated for protein processing and assembly, as well as cell morphogenesis regulation. In the heterosis combination MTP94 × 9475 → MTP67, 7902 overlapping DEGs were significantly enriched in 277 GO terms (FDR <0.05). Similarly, the overlapping DEGs in the MTP67 combination were primarily annotated for biological functions related to protein processing and assembly. The 1622 core DEGs were annotated for biological functions associated with protein processing and assembly and cell morphogenesis regulation (Figure 4H), suggesting that protein processing and folding play a crucial role in establishing heterosis. KEGG pathway mapping of each hybrid combination and the core DEGs revealed that benzoxazinoid biosynthesis, amino acid biosynthesis, protein processing in the endoplasmic reticulum, and cysteine and methionine metabolism were the most enriched pathways (Figure 4I; Tables S11–S13).

In contrast, significantly different functions were identified for the DEGs in the hybrid weakness combinations. In the hybrid weakness combination MTP74 × 9475 → MTP57, 12 628 overlapping DEGs were significantly enriched in 671 GO terms (FDR <0.05). The majority of these genes were functionally annotated with roles related to transmembrane transporter activity, organic hydroxy compound biosynthetic process, response to heat, response to nitrate, photosynthesis, and multiple methyltransferase activities. Among the 10 953 overlapping DEGs from the weakness combination MTP94 × 9475 → MTP66, 456 GO terms were significantly enriched, including response to nitrate, photosynthesis, small molecule metabolism, organonitrogen compound biosynthesis, and photosystem I and II. For the 2940 core DEGs shared by both weakness combinations, the primary annotated biological functions were associated with response to nitrate, photosynthesis, carboxylic acid metabolic process, response to ammonium ion, oxoacid metabolic process, and phosphoenolpyruvate carboxylase activity (Figure 4J). KEGG pathway mapping revealed that alpha‐linolenic acid metabolism, aflatoxin biosynthesis, propanoate metabolism, pyruvate metabolism, ribosome, and carbon metabolism overlapped in each weakness combination and the core DEGs. We hypothesize that changes in the expression of genes involved in these metabolic pathways contribute to hybrid weakness (Figure 4K; Tables S14–S16).

Nevertheless, hybridity is always present in both heterosis and hybrid weakness. Therefore, we analyzed the set of genes common to both the heterosis gene cluster (1622) and the hybrid weakness gene cluster (2940) and observed that 324 genes overlapped between the two clusters (Figure S11a). Functional analysis showed that these 324 DEGs triggered by hybridity were mainly associated with functions including protein folding, negative regulation of hormones, and amino acid metabolism (Figure S11b). The 1298 DEGs triggered exclusively by heterosis were largely related to protein quality control, such as nascent polypeptide‐associated complex, *de novo* cotranslational protein folding, and unfolded protein binding (Figure S11c). DEGs triggered exclusively by hybrid weakness were annotated with functions related to response to nitrate, response to ammonium ions, PEP carboxylase activity, citrate metabolism, phosphoenolpyruvate carboxykinase activity, response to cytokinin, photosynthesis, and carbon fixation (Figure S11d). KEGG pathway enrichment further confirmed these results (Figure S12). The consistent enrichment of protein processing and folding pathways among heterosis‐core DEGs suggests that a reduction in the energetic burden of protein quality control is a fundamental transcriptional signature of heterosis in our allopolyploid system.

### Extensive non‐additive gene expression was observed in perennial forage maize

Expression level dominance (ELD) refers to the phenomenon in which the expression pattern of a hybrid F_1_ resembles that of one of its two parents. To determine the expression level and directionality of allopolyploid trihybrid F_1s_, we classified the DEGs into eight expression patterns according to the method described by Yoo et al. (2013). These patterns include additive (I and XII), maternal‐ELD (IV and IX), paternal‐ELD (II and XI), transgressive downregulation (VII), and transgressive upregulation (VIII) (Figure S13). We selected 99 265 genes with expression levels greater than 1 in at least one plant material for analysis. The results showed that most genes in the trihybrid F_1_ were expressed similarly to one of the parents, although the proportions varied among plant materials. For example, 6.35% (MTP94) to 20.65% (MTP67) of the genes exhibited additive expression, while 22.48% (MTP57) to 62.14% (MTP94) showed maternal‐ELD. In contrast, the proportion of genes exhibiting paternal‐ELD (1.49–29.03%) was significantly lower than that of maternal‐ELD. Additionally, lower percentages of transgressive downregulation (3.78–10.55%) and transgressive upregulation (1.53–13.05%) were observed. Beyond these non‐additive expression patterns, 13.74% (MTP57) to 24.70% (MTP94) of gene expression patterns remained unaltered when comparing the five allopolyploid trihybrids with their parents (Figure S13a).

Further analysis of the proportions of genes exhibiting different expression patterns in the heterosis and hybrid weakness materials revealed that the proportions of genes with unaltered expression in the two heterosis materials, MTP56 and MTP67 (21.35% and 21.09%, respectively), were higher than those in the two hybrid weakness materials, MTP57 and MTP66 (13.74% and 17.56%, respectively). Similar trends were observed for additive expression and maternal‐ELD (Figure S13a). However, this pattern was reversed for parental‐ELD and transgressive expressed genes. For example, the proportion of parental‐ELD in the two heterosis materials, MTP56 and MTP67 (11.69% and 13.00%, respectively), was lower than that in the two hybrid weakness materials, MTP57 and MTP66 (29.03% and 17.45%, respectively). The proportion of transgressive expressed genes in MTP56 and MTP67 (11.61% and 7.58%, respectively) was also lower than that in MTP57 and MTP66 (23.60% and 11.30%, respectively). In contrast to the other four allopolyploid hybrid F_1s_, MTP94 exhibited the lowest percentages of additive expression (6.35%), paternal‐ELD (1.49%), and transgressive expression (5.31%) and the highest percentage of maternal‐ELD (62.14%) (Figure S13a).

The generation of allopolyploid heterosis is typically closely associated with the non‐additive expression of genes. To characterize the functions of these non‐additively expressed genes, six sets of non‐additive genes were collected for KEGG (Figure S13) and GO (Figure S14) enrichment analyses. We observed that the pathways exhibiting high maternal‐ELD in five allopolyploid F_1_ hybrids showed a high degree of concordance. For example, the spliceosome, circadian rhythm, and mRNA surveillance pathways were shared among all F_1s_ (Figure S14b). Regarding the transgressive upregulation expression pattern, the heterotic lines were primarily enriched in pathways related to glyoxylate and dicarboxylate metabolism, carbon metabolism, carbon fixation, starch and sucrose metabolism, amino acid metabolism, and biosynthesis of secondary metabolites. In contrast, the weakness lines were mainly enriched in pathways such as alpha‐linolenic acid metabolism, aflatoxin synthesis, propionate metabolism, thiamine metabolism, and porphyrin metabolism (Figure S14d). Protein processing pathways in the endoplasmic reticulum were enriched in the transgressive downregulation expression pattern across all plant lines (Figure S14g).

### Dosage‐induced DEGs showed a distinct pattern compared to hybridization effects in trihybrids

MTP94 contains two additional complete sets of the *Z. perennis* genome compared to MTP74. Similarly, the differences between the two comparison groups, heterosis materials (MTP67 vs. MTP56) and hybrid weakness materials (MTP66 vs MTP57), involve a difference in one set of *Z. perennis* genome doses. DEGs and their functions were analyzed across the three dosage comparison groups (MTP74 vs. MTP94, MTP56 vs. MTP67, and MTP57 vs. MTP66) (Figure 5). A total of 2222 DEGs were identified in MTP74 vs. MTP94, of which 1454 (65.44%) were upregulated and 768 (34.56%) were downregulated. In the MTP56 vs. MTP67 comparison, 25 296 DEGs were detected, with 11 893 (47.02%) upregulated and 13 403 (52.98%) downregulated. The MTP57 vs. MTP66 comparison revealed 54 628 DEGs, of which 27 504 (50.35%) were upregulated and 27 124 (49.65%) were downregulated (Figure 5A). Evidently, genome dosage changes induced by euploidy (MTP74 vs. MTP94) triggered a narrower range of gene expression modulation compared to the dosage changes caused by aneuploidy (MTP56 vs. MTP67 and MTP57 vs. MTP66). Additionally, 205 overlapping DEGs exhibiting a ‘pure’ dosage effect were observed across all three dosage groups (Figure 5B). GO functional analyses revealed that DEGs associated with aneuploidy and euploidy dosage changes were enriched in distinct functional categories. For instance, the 2222 DEGs related to euploidy dosage changes (MTP74 vs. MTP94) were primarily annotated as catalytic enzymes and macromolecular protein complexes, including ribulose‐bisphosphate carboxylase activity, carbon fixation, and enzyme‐protein complexes involved in ATP synthesis (Figure 5C). The MTP56 vs. MTP67 group was mainly annotated for small molecule metabolic processes, nitrogen compound synthesis, naringenin‐chalcone synthase activity, ADP binding, and CoA carboxylase activity. The MTP57 vs. MTP66 group was predominantly associated with cellular processes, biological regulation, and transmembrane transporter activity. Furthermore, the pure dosage effect DEGs shared among the three dosage comparison groups were chiefly annotated as enzymes and macromolecular protein complexes involved in energy anabolism, such as carbohydrate metabolic, decarboxylase activity, phosphoenolpyruvate‐protein phosphotransferase activity, pyruvate kinase activity, and photosynthesis (Figure 5D). KEGG pathway enrichment analysis of these pure dosage effect DEGs further suggests that pathways related to energy anabolism, including photosynthesis and carbon fixation, may be dosage sensitive (Figure 5D).

**Figure 5:**
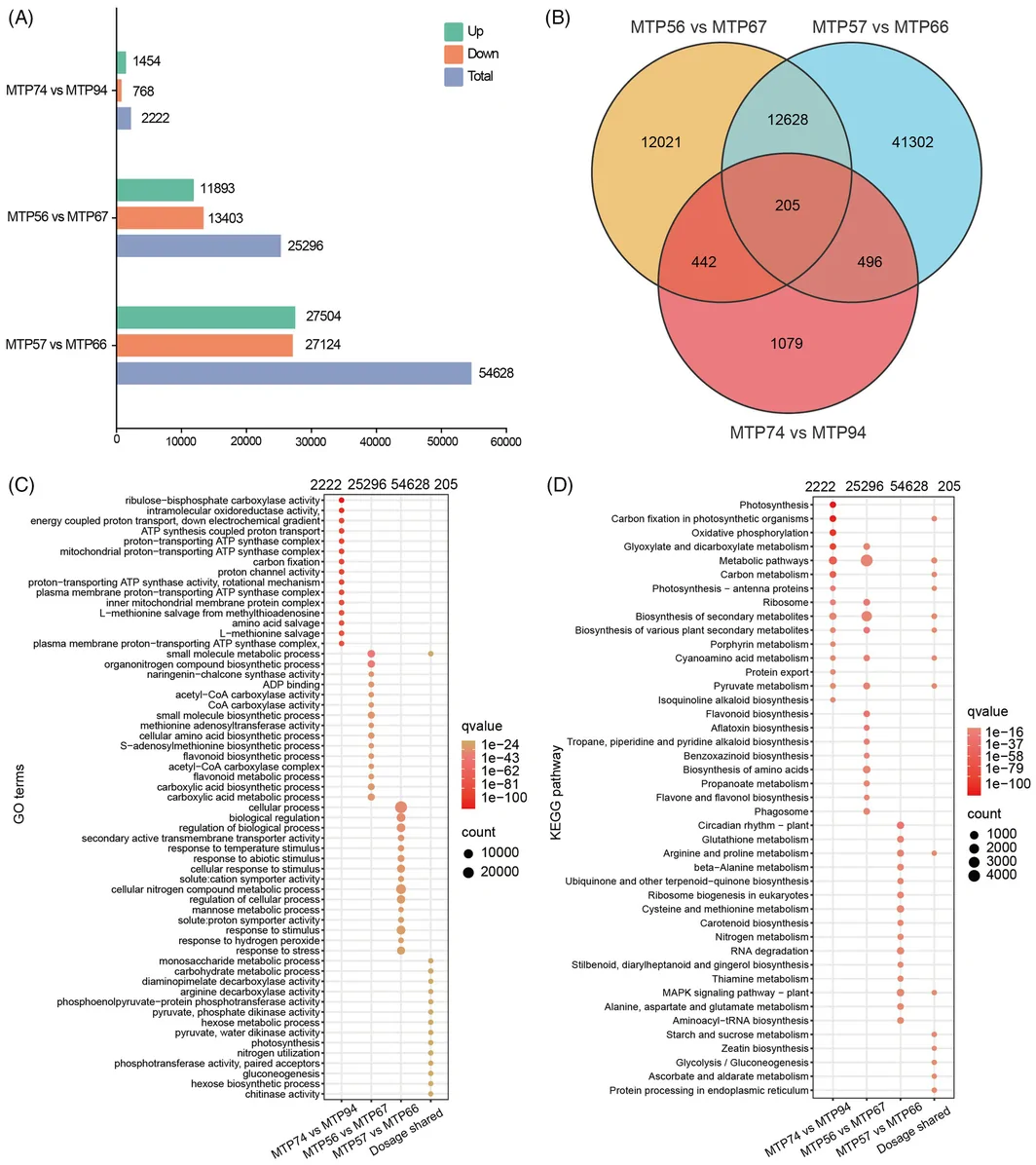
Analysis of DEGs and their functions triggered by genome dosage effects in allopolyploids of perennial forage maize. (A) Overview of the number of DEGs in three genome dosage comparison groups (MTP74 vs. MTP94, MTP56 vs. MTP67, and MTP57 vs. MTP66). (B) Venn diagram illustrating the DEGs associated with the ‘pure’ dosage effect shared among the three dosage comparison groups. (C and D) GO annotations and KEGG pathway enrichment analyses for the three dosage comparison groups and their corresponding ‘pure’ dosage effect DEGs, with the top 10 terms presented for each group.

### WGCNA analysis of hub genes groups associated with heterosis in allopolyploid trihybrids

To move beyond individual DEGs and identify functionally coordinated gene networks underlying heterosis, we performed Weighted gene co‐expression network analysis (WGCNA). This approach enabled us to uncover modules of co‐expressed genes whose collective expression is closely linked to FW heterosis. A total of 1622 overlapping core DEGs from two heterosis comparison groups were analyzed. Seven gene modules were generated, each labeled with a different color in the output (Figure 6A). The largest module (Blue) included 883 genes, while the smallest module (Salmon) contained 51 genes (Figure 6B). The salmon module exhibited the strongest positive correlation with FW (*r* ^2^ = 0.71, *P* = 0.0099) (Figure 6C), indicating that these genes are associated with FW heterosis in the allopolyploid trihybrid. GO annotation analysis showed that genes in the brown module were mainly enriched at 6‐phosphofructokinase activity, diphosphate‐fructose‐6‐phosphate‐1‐phosphotransferase activity, phosphofructokinase activity, protein disulfide isomerase activity, and carbohydrate kinase activity. KEGG pathway enrichment analysis revealed that genes in the salmon module were significantly involved in fructose and mannose metabolism, pentose phosphate pathway, and glycolysis/gluconeogenesis (Figure S15). In addition, the green yellow (containing 58 genes), blue (containing 883 genes), and brown (containing 324 genes) modules exhibited the strongest negative correlation with FW heterosis (Figure 6C). GO annotation analysis revealed that genes in the green‐yellow and brown modules were mainly enriched in protein quality control systems, including the nascent polypeptide‐associated complex, protein folding, *de novo* cotranslational protein folding, and unfolded protein binding. KEGG pathway enrichment indicated that genes in these two modules were significantly involved in protein processing in the endoplasmic reticulum, plant–pathogen interaction, spliceosome function, and ubiquitin‐mediated proteolysis (Figure S15). Collectively, most genes in both the green‐yellow and brown modules participate in the protein quality control system.

**Figure 6:**
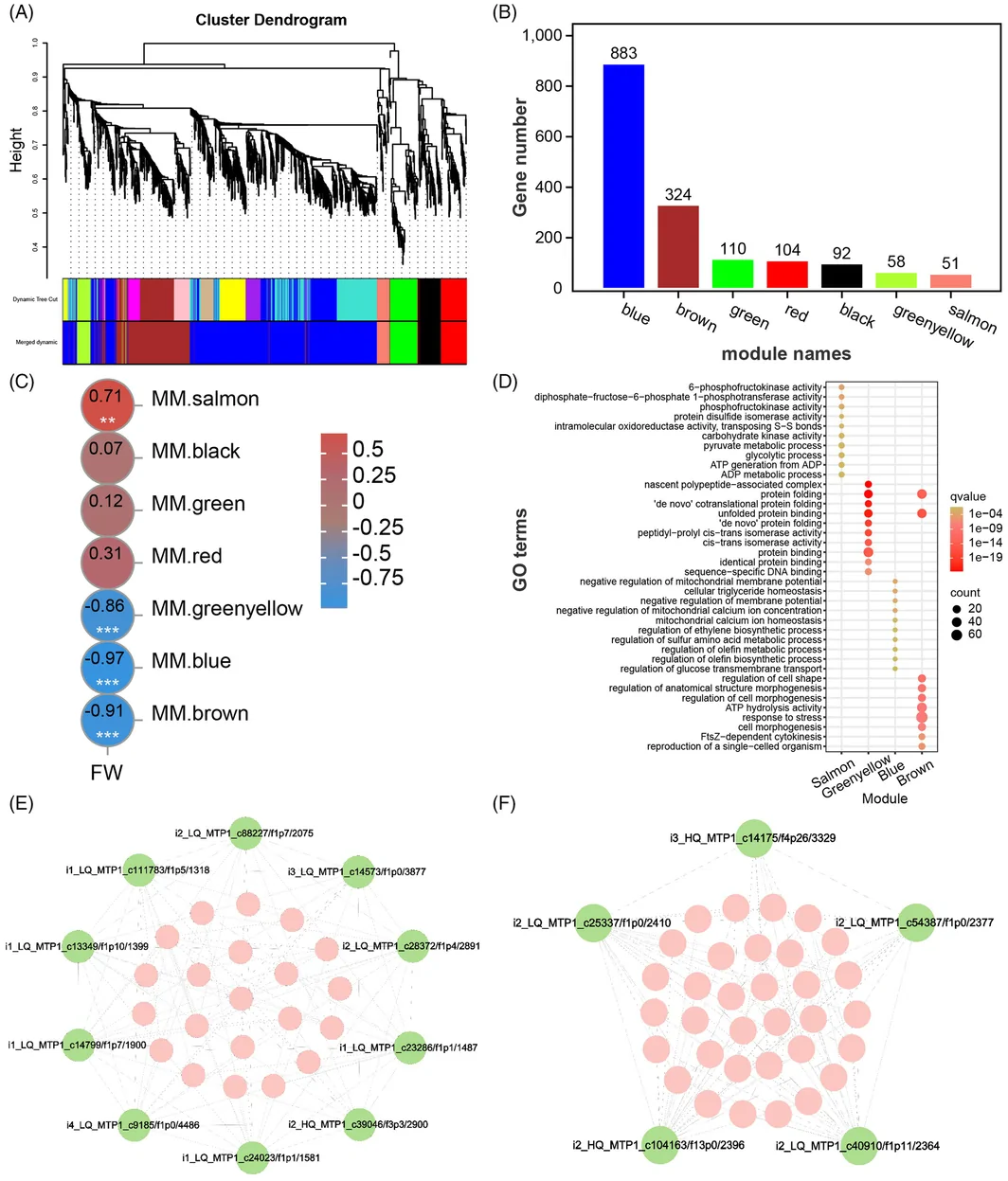
Weighted gene co‐expression network analysis (WGCNA) of overlapping core DEGs from two heterosis comparison groups. (A) Hierarchical cluster tree illustrating gene clustering to divide modules based on a power of *β* = 12. (B) Number of genes in each module. (C) Correlation between each module and fresh weight heterosis in WGCNA. (D) GO functional annotation of genes in four modules significantly associated with fresh weight heterosis. (E) Expression network of genes in the salmon module, highlighting its top ten hub genes. (F) Expression network of genes in the green‐yellow module, highlighting its top five hub genes.

According to functional categories, we classified the enriched pathways significantly associated with biomass heterosis into five types: protein quality control system, glutathione metabolism, spliceosome, energy and glucose metabolism, and metabolic pathways. We observed that genes in the protein quality control system, spliceosome, and glutathione metabolism pathways were lowly expressed in the two heterosis F_1_ hybrids (MTP56 and MTP67) but highly expressed in their parents (MTP74 and MTP94) (Figure S16a–c). Conversely, genes related to energy and sugar metabolism and other metabolic pathways were highly expressed in the F_1_ hybrids but expressed at low levels in their parents (Figure S16d,e). Therefore, we focused further on these four modules, which were significantly associated with FW heterosis to identify hub genes. To this end, Cytoscape software was used to visualize gene co‐expression networks (Figure 6; Figure S16). Ultimately, 10 hub genes were identified in the salmon module (KME >0.90 and weight >0.30) (Figure 6E; Table S17), and five hub genes were identified in each of the green‐yellow, blue, and brown modules (KME >0.90 and weight >0.30) (Figure 6F; Figure S17; Table S17). We infer that these hub genes may play important roles in regulating *Zea–Tripsacum* allopolyploid heterosis; however, their specific functions require further analysis and verification.

### Verification of expression levels of heterosis‐related and dosage effect gene groups in population materials

To validate the expression levels of genes within modules significantly associated with FW heterosis at the population level, we selected 10 genes from modules that were significantly positively correlated with FW heterosis and compared them to 10 genes selected from three modules that were significantly negatively correlated. In pop79 and pop99, we selected 10 heterotic lines and compared them to 11 disadvantageous lines. Additionally, we included diploid maize inbred lines Mo17, B73, and their hybrid Mo17 × B73 as controls. The results showed that among the 10 genes in the module significantly positively correlated with FW heterosis, nine exhibited higher average expression levels in the heterotic lines than in the disadvantageous plant lines in both populations (Figure 7). Conversely, the 10 genes in the module significantly negatively correlated with FW heterosis showed higher average expression levels in the disadvantageous lines than in the heterotic lines in both populations (Figure S18). Furthermore, validation of the expression levels of 205 dosage‐responsive DEGs across 20 accessions (including both pop79 and pop99) revealed that materials with higher genomic dosage exhibited elevated expression levels, regardless of whether they were classified as superior or inferior accessions (Figure S19). These results further support the existence of dosage effects.

**Figure 7:**
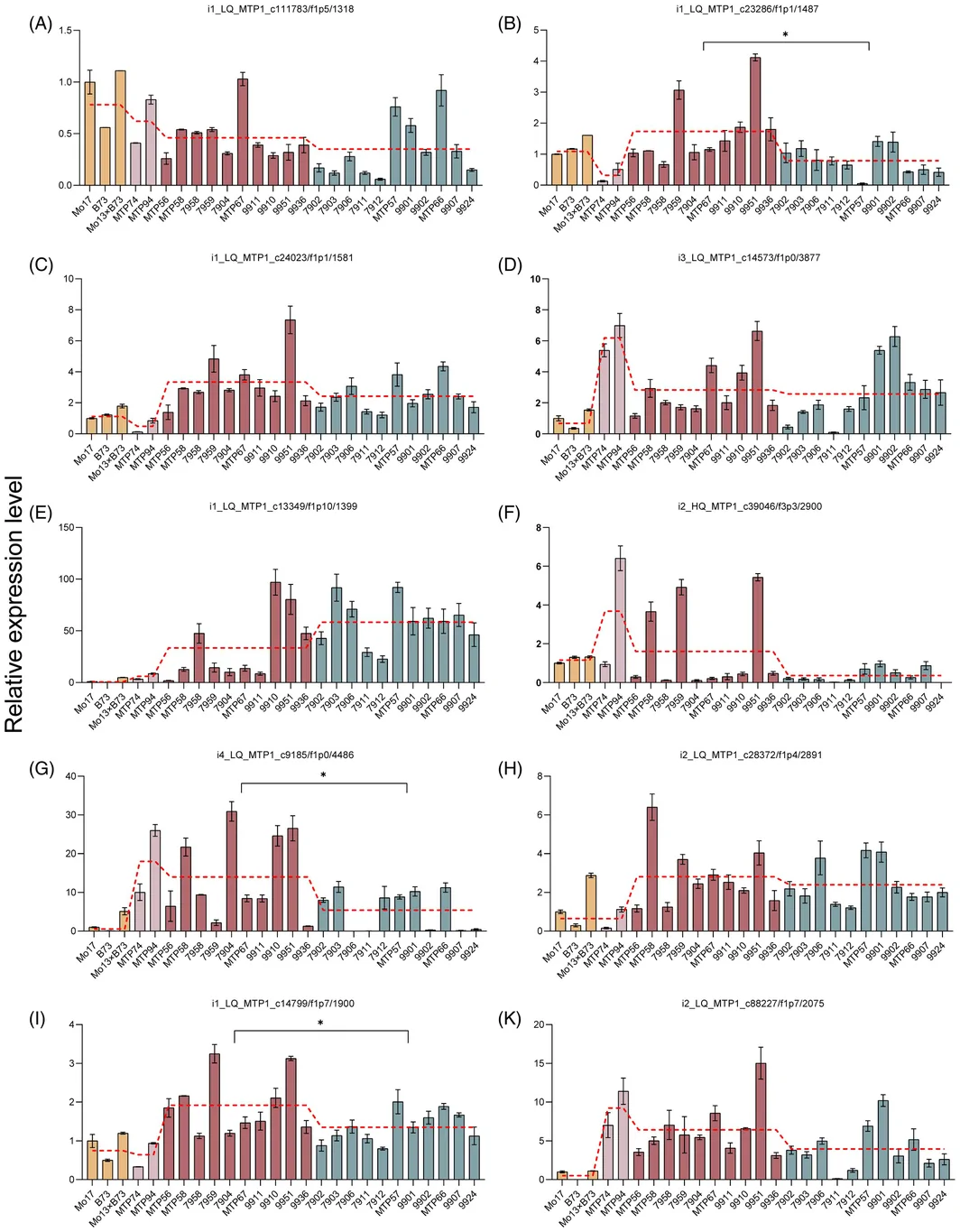
Validation of gene expression in salmon module at population level. (A–K) represent the expression levels of 10 genes in the module positively correlated with fresh weight heterosis at the population level. In the figure, the yellow, pink, and wine‐colored columns represent diploid maize, polyploid parent materials, and heterosis plant materials, respectively, while the blue columns represent weakness plant materials. The red dashed line represents the overall average. An asterisk denotes that the average expression level of all superior lines is significantly higher (*P* < 0.05) than that of hybrid weakness lines.

### Perennial forage maize lines developed via genome shuffling: Harnessing heterosis, genomic dosage, and perenniality

By integrating heterosis, genomic dosage effects, and perenniality through genome shuffling, we developed two perennial forage maize lines that outperform annual silage maize across multi‐site trials. The allohexaploid MTP74 (2*n* = 6*x* = 74) was first synthesized in 2006 by crossing *Z. mays*, *T. dactyloides*, and *Z. perennis*. Subsequent crosses with *Z. perennis* produced the pop79 population, from which the national cultivars Yucao 5 and Yucao 6 were released; cumulative adoption exceeds 100 000 hectares, with regrowth persisting for more than seven. The alloctoploid MTP94 (2*n* = 8*x* = 94) was then generated via 2*n*+*n* sexual polyploidization (MTP74 × *Z. perennis*). Crossing MTP94 with tetraploid perennial teosinte yielded pop99, which gave rise to Yucao 9911 and Yucao 9919. Both populations exhibit perenniality and facultative apomixis, constituting usable germplasm for dissecting heterosis and dosage effects.

In the pop79 and pop99, we identified two lines, MTP56 (released as Yucao 6) and MTP67 (released as Yucao 9919), which exhibited high single‐plant yields (11 kg plant^−1^ and 14 kg plant^−1^, respectively), favorable plant architecture (Figure 2a, b), and 100% overwinter ability in Chengdu. These lines were officially validated as new forage varieties by agricultural authorities in 2018 and 2019, respectively. Over four regrowth cycles, Yucao 6 produced an average annual fresh yield of 127.7 t ha^−1^ (24.1 t hay), while Yucao 9919 achieved 142.6 t ha^−1^ (26.5 t hay), both significantly surpassing the elite annual control, silage corn Yayu 8 (49.8 t fresh; 15.5 t hay). This yield advantage was consistent in Chengdu and Nanchong; although elevation‐related winter stress in Liangshan reduced the advantage. Yucao 9919 demonstrated enhanced mowing tolerance and cold resistance, attributed to an extra *Z. perennis* genome dosage (Tables S18–S20). These findings demonstrate that genome‐shuffling‐mediated pyramiding of heterosis, dosage effects, and perenniality can generate commercially viable forage maize, providing a transformative option for sustainable feed production in hilly subtropical regions.

## DISCUSSION

### Superior plant traits typically represent an integrated manifestation of both heterosis and genomic dosage effects

In plants, superior performance typically arises from two distinct genetic mechanisms: heterosis derived from hybridization (Birchler et al., 2010; Hochholdinger & Yu, 2025) and dosage effects caused by genome accumulation or alteration (Bastiaanse et al., 2019, 2021; Benoit et al., 2025; Henry et al., 2015; Yao et al., 2013; Zhang et al., 2025). However, aneuploidy (chromosome gain or loss) is generally considered deleterious, as it causes genetic imbalance and reduced fitness (Birchler & Veitia, 2007). Nevertheless, emerging evidence from nascent polyploids challenges this view by demonstrating that intergenomic chromosome combinations can compensate for the negative impacts of aneuploidy on growth and adaptive potential. In synthetic allotetraploid wheat, Wang et al. (2026) found that approximately 30% of transgenerationally selected aneuploid cohorts exhibited higher progeny fitness than euploid counterparts even under normal conditions, with specific aneuploidies (e.g., trisomy 2A/monosomy 2D) predominating among stress survivors. In nascent allohexaploid wheat, Zhang et al. (2013) documented persistent whole‐chromosome aneuploidy, including a ‘hidden’ type that maintaining 2*n* = 42 through simultaneous loss and gain of non‐homologous chromosomes. In the natural allotetraploid *Tragopogon miscellus*, Chester et al. (2012) reported that 69% of individuals were aneuploid; however, 85% of these were compensated aneuploids, preserving the total chromosome number. Building upon our previous and ongoing investigations, we have successfully achieved genome amalgamation among *Z. mays*, *Z. perennis*, and *T. dactyloides* through 2*n*+*n*‐mediated sexual polyploidization (Iqbal et al., 2019; Li et al., 2024; Yan, Cheng, et al., 2020). Using MTP74, MTP94, and MTP58 as maternal parents in backcrosses with *Z. perennis*, we generated three distinct populations (pop79, pop99, and pop59) (Li et al., 2024). Our study demonstrated that aneuploidy is pervasive across these populations, with hypoploidy predominating (85.7% of individuals), yet many lines exhibited vigorous growth. Notably, we observed a genome balance mechanism between maize and *Z. perennis*, despite wide variation in individual chromosome numbers, the combined Zm + Zp count remained stable, suggesting functional compensation. Furthermore, the lowest‐ploidy population pop59 showed significantly reduced phenotypic fitness compared to pop79 and pop99, indicating that intergenomic chromosome combinations and elevated ploidy levels can partially mitigate the deleterious effects of aneuploidy on vegetative growth (Li et al., 2024).

Vegetative vigor and reproductive success are not equivalent. In our MTPs, aneuploidy and intergeneric hybridization were compatible with strong vegetative heterosis, as measured by fresh weight, plant height, tiller number, and photosynthetic capacity. However, reproductive traits did not always exhibit parallel heterosis (Figure S2), and pollen viability was extremely low (<2%; Figures S20–S22). This indicates that the genomic and dosage perturbations that enhance vegetative growth can be uncoupled from those required for meiosis, gametogenesis, and seed set. However, our breeding strategy does not rely on seed propagation to maintain these materials. Sexual reproduction generates heterosis and genome dosage advantages, while vegetative propagation fixes and multiplies these traits as clonal lineages without altering the original genotype. Therefore, low pollen fertility is not a barrier to achieving high forage yield or multi‐year regrowth in vegetative propagation or perennial plants. In contrast, grain crops require additional strategies to restore fertility and meiotic stability while preserving dosage‐mediated vigor.

Analysis of hybrid vigor in the two populations revealed that, while individual biomass varied significantly, the populations exhibited pronounced heterosis. Despite similar chromosomal dosages and compositions among progeny, substantial phenotypic variation was observed in key agronomic traits such as yield. Notably, accessions with fewer chromosomes, including MTP58, MTP56, and MTP67, demonstrated superior performance compared to their higher‐ploidy counterparts (MTP74 and MTP94). This observation challenges the conventional notion that hybrid vigor results solely from chromosomal or genomic accumulation, suggesting instead that heterosis may arise from complex intergenomic interactions and regulatory coordination triggered by hybridization. The intricate and often unpredictable nature of hybrid performance, encompassing both heterosis and hybrid weakness, underscores the complexity of these genetic interactions. In contrast, dosage effect analysis revealed a more predictable pattern. From MTP74 to MTP94 and across the pop79 to pop99 populations, a positive trend between chromosomal dosage and yield advantage was observed. This dosage‐dependent enhancement of agronomic performance supports the concept of ‘more is better’ in the context of optimal genomic dosage, where appropriate increases in chromosome number or gene dosage may confer beneficial effects.

Any comprehensive theory of heterosis must reconcile the paradoxical observation that, while heterosis levels in maize hybrids have remained static or slightly declined since 1930, consistent yield improvements in both inbred lines and hybrids have been documented (Duvick, 1992, 1999; Troyer & Wellin, 2009). This suggests an increasingly dominant role of inbred line improvement in driving hybrid yield gains. Our findings further demonstrate that superior performance in hybrids with complex genomes represents a synergistic integration of heterosis and genomic dosage effects, with the latter constituting a distinct genetic advantage. Specifically, comparative analysis revealed equivalent heterosis levels between the pop79 (MTP74 × 9475) and pop99 (MTP74 × 9475) hybrid populations. However, a progressive yield enhancement was observed with increasing genomic dosage from MTP74 to MTP94 across the corresponding hybrid populations. This dosage‐dependent yield amplification aligns with Duvick's paradigm (Duvick, 1992), wherein hybrid superiority arises not solely from heterosis but also from the cumulative dosage effects of elite alleles in parental lines (Sun et al., 2023; Yao et al., 2013). Our findings highlight the increasing importance of cumulative dosage effects in driving yield improvement, suggesting that targeted manipulation of genomic dosage could complement traditional heterosis‐based approaches to achieve the next generation of high‐performance hybrids.

### Functional divergence caused by hybridization and genome dosage

Utilizing high‐throughput transcriptomic analyses to profile gene expression patterns in complex neopolyploids provides critical insights into the shared and distinct regulatory mechanisms underlying heterosis, hybrid weakness, and dosage effects across multiple biological levels, including gene expression dynamics, functional pathway modulation, and network architecture reorganization. Previous studies have demonstrated that heterosis can be triggered by genomic or gene dosage effects, particularly in polyploid organisms that possess varying degrees of genome dosage (Fort et al., 2016; He et al., 2022; Washburn & Birchler, 2014; Yao et al., 2013). Allopolyploids are, by definition, ‘hybrids’ in terms of their formation and the origin of subgenomes (Chen, 2007, 2010; Fort et al., 2016). Therefore, unlike diploids, allopolyploids experience heterosis triggered not only by heterozygosity or hybridity, but also by genome doubling, which confers dosage effects. However, distinguishing the effects of genome dosage and hybridity on gene expression, as well as their functional similarities and differences in an allopolyploid system, remains challenging. Previous studies of gene expression in polyploids across different species have shown that transcriptome shocks triggered by interspecific hybridization can be ameliorated by genome doubling, although the extent of this amelioration depends on species variability (Buggs et al., 2011; Hao et al., 2017; Hegarty et al., 2006; Qin et al., 2021; Wang et al., 2006). For example, in Arabidopsis polyploids, Wang et al. (2006) observed that 1362 and 1469 genes exhibited expression divergence in two independently derived synthetic allotetraploids relative to mid‐parent values, suggesting that these non‐additive regulations are triggered by hybridization. In contrast, only 88 genes were significantly differentially expressed between *A. thaliana* diploids and the isogenic autotetraploid. Although the microarray technique used in this study may have underestimated the number of DEGs between the allotetraploid and the parents, it still provides valuable insights. In synthetic triploid wheat, Hao et al. (2017) observed that the abundance of homoeologous genes was disrupted by hybridization, but this disruption was partially restored after synthesizing an allohexaploid via genome doubling. Additionally, studies in *Senecio* and *Spartina* have shown that hybridization‐induced transcriptome shock can be mitigated by genome duplication (Banouh et al., 2023; Chelaifa, Mahe, & Ainouche, 2010; Chelaifa, Monnier, & Ainouche, 2010; Hegarty et al., 2006; Salmon et al., 2005). In this study, extensive transcriptome reprogramming was observed in synthesized allopolyploid trihybrids through hybridization remodeling of MTP74 and MTP94. For example, 18 436, 29 068, 65 333, and 41 437 DEGs were identified in comparisons of MTP74 vs. MTP56, MTP94 vs. MTP67, MTP74 vs. MTP57, and MTP94 vs. MTP66, respectively. In contrast, MTP94 was synthesized by adding two complete *Z. perennis* subgenomes to MTP74 via 2*n*+*n* sexual polyploidization, and only 2222 genes were significantly differentially expressed between MTP94 and MTP74. However, tens of thousands of DEGs were observed in two additional analyses comparing aneuploidy dosage group (MTP56 vs. MTP67 and MTP57 vs. MTP66), even though the dosage difference primarily involved a set of *Z. perennis* subgenomes. Previous studies have shown that altering the complete genome (ploidy) has a much smaller effect on phenotype and gene expression modulation than altering a single or a few chromosomes (aneuploidy) (Guo et al., 1996; Hou et al., 2018; Shi et al., 2021; Yang, Shi, et al., 2021). This phenomenon can be explained by the Gene Balance Hypothesis (GBH), which states that varying the stoichiometry of members of multisubunit complexes can affect their kinetics and the amount of functional complete product, ultimately leading to negative fitness consequences (Birchler & Veitia, 2007, 2012; Veitia & Birchler, 2022).

In fact, in studies related to the analysis of synthetic allopolyploid heterosis through gene expression profiling, an area that has received little attention is the extent to which the functional and pathway classes involved in DEGs resulting from hybridization and genomic dosage changes actually differ. For example, in DEG analysis of *Brassica napus* hybrid F_1_ and its parents, early expression of genes related to photosynthesis and auxin biosynthesis in the hybrid contributed to heterosis (Zhu et al., 2020). Similar studies in cotton revealed that the function of DEGs is tissue specific; for example, the GO terms for DEGs in leaves primarily involve chloroplast, membrane, and protein binding, whereas in roots, they relate to plasma membrane, extracellular region, and responses to stress (Shahzad et al., 2020). Transcriptional analysis of pigeonpea heterosis indicates that genes related to plant immunity and growth are overexpressed, whereas DEGs associated with basal defense levels are repressed in hybrids (Sinha et al., 2020). Other studies across different species have revealed that genes involved in photosynthesis, cell cycle, energy metabolism, hormone synthesis, and circadian rhythms as contributors to heterosis (Ko et al., 2016; Li, Lu, et al., 2021; Li, Su, et al., 2021; Liu, He, & Deng, 2021; Yang, Liu, et al., 2021). Notably, a recent high‐resolution time‐series transcriptomic analysis in Arabidopsis demonstrated that complementary high‐parent expression of network hub genes involved in the cell cycle and photosynthesis acts as a key driver of biomass heterosis (Liu, He, & Deng, 2021). In the present study, the core DEGs shared by the two heterosis combinations are mainly associated with benzoxazinoid biosynthesis, biosynthesis of amino acids, protein processing in endoplasmic reticulum, cysteine and methionine metabolism, synthesis of secondary metabolites, photosynthesis‐antenna proteins, and flavonoid synthesis pathways. In contrast, the core DEGs shared by the hybrid weakness combinations mapped to putative pathways mainly involving nitrogen metabolism, glutathione metabolism, and porphyrin metabolism. Phenotypic observations further showed that, compared with heterotic materials, leaves of hybrid weakness materials were more susceptible to disease, tended to be chlorotic, and exhibited lower chlorophyll content and photosynthetic rates, implying that impaired nutrient uptake and photosynthesis may underlie hybrid weakness. These results suggest that hybrid weakness may involve abnormal activation of immune pathways leading to growth inhibition (Yang, Liu, et al., 2021). These results are consistent with previous studies. Epigenetic repression of *CCA1*/*LHY* in Arabidopsis hybrids and allopolyploids upregulates chlorophyll and starch pathways and increases biomass (Ni et al., 2009); hybrids also repress stress‐responsive genes under normal conditions, induce them only when necessary, thereby balancing growth and defense (Miller et al., 2015); maize hybrids optimize photosynthetic versus photorespiratory metabolism in a diel manner, enhancing carbon gain and reducing toxic photorespiratory intermediates (Li et al., 2020). Our heterosis‐core DEGs, enriched in protein quality control and accompanied by upregulated energy and carbon metabolism, may represent a transcriptional signature of reduced protein turnover and enhanced carbon assimilation.

When hybrid weakness and heterosis combinations were analyzed together to extract functional pathways of hybridization‐triggered genes, the enriched terms focused on protein folding, negative regulation of hormones, and amino acid metabolism. Conversely, the functional terms of the genome dosage altered‐triggered DEGs in the dosage comparison group mainly involved pathways related to photosynthesis, carbon fixation, oxidative phosphorylation, glyoxylate and dicarboxylate metabolism, ribosome, secondary metabolites, and porphyrin metabolism. Previous studies on duplicate gene retention patterns following polyploidization have shown that the transcription factor, kinase exhibit a higher retention rate (Blanc & Wolfe, 2004; Maere et al., 2005). Photosystem related genes, such as those encoding photosystem I and photosystem II components, have been observed to exhibit dosage sensitivity in *Glycine max* and *Medicago truncatula* polyploid organisms (Coate et al., 2011). In addition, expression level‐based studies have identified specific GO terms exhibiting gene dosage sensitivity, including ATP hydrolysis coupled proton transport, protein phosphatase type 2A complex, protein modification, ubiquitination, oxygen evolving complex, signal transduction, and protein serine/threonine kinase activity (Coate et al., 2016). Furthermore, in, RNA‐seq investigations of maize aneuploid series revealed distinct dosage sensitivities among functional gene classes, including transcription factors (TFs), signal transduction components, ribosomal subunits, proteasomal machinery, and nuclear‐encoded chloroplast‐ and mitochondrial‐targeted genes. These classes exhibited non‐linear expression modulation patterns, with the direction and magnitude of responses contingent on both the extent of chromosomal imbalance (aneuploidy) and whole‐genome ploidy shifts. For instance, ribosomal and chloroplast‐targeted genes displayed pronounced inverse trans‐modulation in hyperploid aneuploids, while TFs and signaling components showed subtler yet significant stoichiometric shifts. Notably, ploidy changes induced minimal global expression variation compared to aneuploidy, underscoring the heightened sensitivity of regulatory networks to segmental dosage alterations. These findings align with kinetic models of multicomponent interactions, where stoichiometric imbalances disrupt assembly dynamics, thereby amplifying phenotypic and transcriptomic consequences in a dosage‐dependent manner (Shi et al., 2021; Yang, Shi, et al., 2021). In this study, the functional categories of the overrepresented DEGs between the dosage comparison groups were essentially consistent with those reported in previous studies. Overall, this study deepens our understanding of the molecular pathways affected by hybridization and genomic dosage changes in polyploid organisms.

### The role of protein stability and quality control systems in the formation of heterosis

The importance of heterosis in agriculture requires no further explanation. However, the mechanisms underlying heterosis are much less studied and understood than its practical applications. Several popular genetic models have been proposed to explain heterosis, including those involving dominance (Bruce, 1910; Jones, 1917), overdominance (Crow, 1948; East, 1936), and epistasis (Schnell & Cockerham, 1992; Yu et al., 1997). Nevertheless, there is no single model that adequately explains how heterosis is generated. To unify the theory of heterosis, researchers have proposed hypotheses based on metabolic and energy use efficiency (Ginn, 2010, 2017; Goff, 2011; Goff & Zhang, 2013). Previous studies have demonstrated that inbred organisms typically have higher protein turnover rates compared to non‐inbred organisms (Bayne, 2004; Hawkins et al., 1986; Hedgecock et al., 1996). Through protein molecular dynamics simulations and theoretical calculations, it has been proposed that elevated protein turnover in inbred organisms may result from the accumulation of misfolded and aggregated proteins, which require degradation by the protein quality control system (Ginn, 2010, 2017). Since protein synthesis and degradation are energy‐consuming processes, inbred organisms must consume more energy to maintain a given biomass and are therefore less metabolically efficient. It is speculated that hybrids exhibit more efficient growth compared to their corresponding inbreds due to differences in protein metabolism (Goff, 2011). This efficiency likely arises because the presence of multiple distinct alleles at a single gene locus in hybrids allows for the preferential expression of the more stable allele. Thus, hybrids and allopolyploids possess allelic diversity, providing more opportunities for selective expression and thereby increasing heterosis (Goff, 2011).

Our transcriptional profiling supports the energy use efficiency hypothesis (Ginn, 2010, 2017; Goff, 2011; Goff & Zhang, 2013). For example, we observed that the overrepresentation of DEGs triggered by hybridization in both heterosis and hybrid weakness combinations was predominantly enriched in the protein processing in endoplasmic reticulum pathway. The downregulation of genes associated with protein quality control systems in the heterotic F_1_ trihybrids (MTP56 and MTP67), which was reversed in their female parents (MTP74 and MTP94), suggests a lower protein turnover rate and accumulation of polymers in the F_1_ trihybrids compared to the female parent lines. In addition, we noted high expression levels related to energy and glucose metabolism, as well as basic metabolic pathways in F_1_ trihybrids. Consistent with this, downregulation of genes involved in protein quality control systems has been observed in *Drosophila* hybrids, where misfolded and aggregated proteins increase the metabolic efficiency of the hybrids (Kristensen et al., 2002; Pedersen et al., 2005). A previous study also showed that upregulation of genes related to one‐carbon metabolism and downregulation of genes for protein quality control systems in hybrids contribute to yeast heterosis at high temperature (Song et al., 2021). Recent studies in Arabidopsis have demonstrated that the solubility of proteins related to ribosome biosynthesis, thylakoid lumen, membrane, and stroma contributes to heterosis in Arabidopsis hybrids and allotetraploids (June et al., 2023). Therefore, our data support a model in which the allelic diversity introduced by hybridization allows for the selection of more stable protein variants, reducing the load on the protein quality control system. The energy saved can then be reallocated to growth and biomass accumulation, manifesting as heterosis. This provides a mechanistic link between the classic genetic models of heterosis and the physiological outcome of enhanced metabolic efficiency.

### Integrated exploitation of heterosis and dosage effects in perennial forage maize breeding

Hybrid‐breeding and the exploitation of heterosis have significantly contributed to the sustained increase in agricultural production (Mackay et al., 2021). However, several studies have demonstrated the non‐negligible role of genomic dosage components in the formation of plant advantages, in both diploid and polyploid contexts. For instance, in maize diploid hybrid F_1_ and triploid hybrid heterosis in a diploid background shows M^*a*^M^*a*^ < M^*a*^M^*b*^ ≈ M^*b*^M^*a*^ > M^*b*^M^*b*^, but significant differences were observed between reciprocal triploid hybrids, such as M^*a*^M^*a*^M^*a*^ < M^*a*^M^*a*^M^*b*^ ≠ M^*b*^M^*b*^M^*a*^ > M^*b*^M^*b*^M^*b*^ (Yao et al., 2013). Progressive polyploid heterosis effects have been documented across potato (Mok & Peloquin, 1975), banana (Batte et al., 2020), *alfalfa* (Bingham et al., 1994; Groose et al., 1989), tomato (Wang et al., 2024), and maize (Washburn et al., 2019; Washburn & Birchler, 2014). Specifically, Heterosis in maize tetraploid double‐cross F_1_ (ABCD) exceeds that of tetraploid monohybrid hybrids (AABB and CCDD). The progressive heterosis observed in potato is attributed to higher allelic diversity in tetraploids compared to diploids, resulting in epistatic effects (Jansky, 2009). These studies demonstrate that t genome dosage composition is closely related to heterosis. Moreover, parent‐of‐origin effects on genome dosage influencing heterosis have been demonstrated at the polyploid level. For example, in *A. thaliana* ploidy series generated by hybridization and genomic dosage remodeling, F_1_ triploids with two paternal genomes display positive heterosis in seed size and leaf area, whereas F_1_ triploids with two maternal genomes show negative heterosis effects (Fort et al., 2016). Collectively, these findings establish that genome dosage composition is intimately linked to heterotic outcomes.

Understanding and manipulation of plant reproduction are crucial for crop breeding and germplasm innovation (Iqbal et al., 2019). Polyploid formation often involves mechanisms such as unreduced gamete formation (Brownfield & Kohler, 2011), somatic chromosome doubling (Van de Peer et al., 2017), polyspermy fertilization (Toda & Okamoto, 2020), and asexual reproduction (Freeling, 2017). Notably, the apomictic reproductive traits of wild species can be harnessed to adjust genomic dosage and fix plant vigor (dosage benefit and heterosis) to improve existing cultivars. For example, a recent study reported the synthesis of interspecific hybrids by incorporating the *Pennisetum squamulatum* genome into *P. glaucum* via apomixis. These hybrids exhibited significant trait variation and heterosis, along with enhanced nutritional quality, and this heterosis could be permanently fixed (Roy et al., 2022).

In our systematic work, MTP74 was used as a parental line in crosses and backcrosses with its parents to develop novel maize germplasm and perennial forage maize (He, Yang, et al., 2023; He, Zheng, et al., 2023; Iqbal et al., 2019, 2023; Li et al., 2024; Zhou et al., 2025), MTP74 exhibited four reproductive modes, including fertilization of an unreduced gamete (2*n*+*n*), apomixis (2*n*), normal chromosome reduction (*n*+*n*), and apomictic (*n*) (Iqbal et al., 2019, 2023; Li et al., 2024). This reproductive diversity likely stems from the apomictic characteristics inherited from one of its parents, *T. dactyloides* (Burson et al., 1990; Grimanelli et al., 1998), whereas the other parent, *Z. perennis*, never exhibits apomictic potency (Iqbal et al., 2019). In the present study, based on the allohexaploid MTP74, we further synthesized the allooctaploid MTP94 by adding two sets of *Z. perennis* subgenomes to MTP74 via apomictic reproduction. Furthermore, pop79 and pop99 were synthesized by crossing the highly ploidy MTP74 and MTP94, respectively, as female parents with *Z. perennis* to remodel the genomic dosage of these two female parents. Briefly, this strategy effectively released the ‘frozen’ variability in the two apomictic species, MTP74 and MTP94, by utilizing the sexuality of *Z. perennis*. Through systematic evaluation of elite trihybrids, MTP56 and MTP67, derived from pop79 and pop99, across diverse ecoregions, we developed and commercialized a series of groundbreaking perennial forage maize cultivars, such as Yucao 6 and Yucao 9919. These cultivars exhibit superior agronomic performance, characterized by enhanced productivity (dry matter yield ranging from 24.08 to 26.54 t ha^−1^), high nutritional quality (whole‐plant crude protein content exceeding 10%) (Li et al., 2019). Newly synthesized polyploids often face challenges such as reduced genetic diversity and meiotic disorders when reproducing sexually (Freeling, 2017). A viable strategy for long‐term survival of new polyploids is to protect the reproductive system by circumventing meiosis (Freeling, 2017). Asexual vegetative reproduction, which is widespread among plants, is typically associated with perenniality and polyploidy (Freeling, 2017). All *Zea*–*Tripsacum* trihybrids generated in our study can be perennialized via nodal and rhizome propagation. Multi‐year field demonstrations confirm that these cultivars sustain regrowth at least five to 7 years without sudden yield decline. Additionally, the aneuploidy in these cultivars causes low pollen viability (<2%) (Figures S20–S22), minimizing unwanted spread and addressing ecological concerns. These cultivars have been promoted on over 10 thousand hectares in southern China (Figure S23). Overall, this strategy generates abundant diversity through interspecific hybridization, 2*n*+*n* genome doubling, and chromosomal recombination, while fixing heterosis and genome dosage vigor via perenniality and vegetative propagation. It offers a practical, efficient breeding route applicable to forage crops and other polyploid or clonally propagated species like sugarcane, potato, and bioenergy grasses.

## MATERIALS AND METHODS

### Plant materials

Tetraploid maize (accession V182, 2*n* = 4*x* = 40Zm) was crossed as the female parent with tetraploid *T. dactyloides* (accession TP209, 2*n* = 4*x* = 72Td), and embryos were rescued to obtain maize‐*Tripsacum* hybrid (accession H278, 2*n* = 56 = 20Zm + 36Td), as detailed in previous studies (Kindiger, Sokolov, & Dewald, 1996; Kindiger, Sokolov, & Khatypova, 1996). The allohexaploid trihybrid MTP74 (2*n* = 74 = 20Zm + 34Td + 20Zp) was synthesized via 2*n*+*n* sexual polyploidization, involving an unreduced 2*n* female gamete (2*n* = 20Zm + 36Td) from abnormal meiosis of H278 crossed with a normal *n* male gamete (*n* = 20Zp) from *Z. perennis* (accession 9475, 2*n* = 4*x* = 40Zp) (Iqbal et al., 2019; Li et al., 2024; Yan, Cheng, et al., 2020). Subsequently, the allooctaploid MTP94 (2*n* = 94 = 20Zm + 34Td + 40Zp) was produced by crossing the MTP74 with *Z. perennis* via 2*n* *n* polyploidization. *Z. perennis* was then used as the male parent to cross with the MTP74 and MTP94, generating the pop79 and pop99 populations, respectively (Li et al., 2024).

### Karyotyping using genome *in situ* hybridization (GISH)

Seedling cultivation and chromosome preparation methods followed Yan, Li, et al. (2020) and Li et al. (2024). The GISH pipeline was conducted following the protocol described by Iqbal et al. (2019). *Z. mays* and *Z. perennis* genomic DNAs were labeled with Dig‐ and Biotin‐Nick Translation Mixes (Roche), respectively, per manufacturer instructions. Fluorescence images were captured using an Olympus BX61 microscope with a 100× Plan Apochromat oil immersion objective and a Media Cybernetics CCD 700 camera. Image acquisition and initial processing used Image Pro Plus 6.0 (Media Cybernetics) on Windows. Pseudocolors were assigned as follows: DAPI (Vector Laboratories) blue, *Z. mays* probe green, and *Z. perennis* probe red. Multichannel images were merged in the same software. Final adjustments—cropping, resizing, and contrast optimization—were applied globally using Adobe Photoshop CC 19.1.6, with all images exported as uncompressed TIFF files.

### Plant growth conditions and morphological measurement

The pop79 and pop99 populations, along with their parental lines, were cultivated at the Chongzhou Experimental Base of Sichuan Agricultural University (30°33′ N, 103°38′ E, altitude 523 m). Due to the tall grass‐type morphology of the MTPs polyploid materials, plants were spaced 1.20 × 1.50 m. Uniform seedlings with a single tiller were selected and transplanted at a depth of 5–10 cm, with nine plants per genotype. After planting, thorough irrigation ensured root establishment. Weed control was manual, and other field management followed standard agronomic practices. Additionally, tetraploid *Z. perennis*, core maternal lines (MTP74, MTP94), representative heterosis lines (MTP56, MTP67), and hybrid weakness lines (MTP57, MTP66) were grown at the Wenjiang Experimental Base (30°72′ N, 103°87′ E, altitude 420 m). Leaves were trimmed before planting, with 18 plants per genotype arranged in rows for growth dynamics, microscopic trait assessment, and photosynthetic measurements, supporting transcriptome sampling and analysis.

Yield and agronomic traits were measured following established protocols (Li et al., 2024). Phenotypic parameters included plant height (PH, cm), tiller number (TN), leaf length (LL, cm), leaf width (LW, cm), leaf area (LA, cm^2^), leaf number (LN), stem diameter (SD, mm), spike length (SP, cm), kernel number per row (KNPR), tassel length (TL, cm), tassel branch number (TBN), silk length (SL, cm), silk diameter (SKD, μm), and fresh weight per plant (FW, kg·plant^−1^). Measurements were taken at 105 days after sowing (DAS) with at least three biological replicates per genotype.

### Stomatal and pollen size measurement

For stomata analysis, leaves from the middle section of the first functional leaf of each genotype were collected between 9:00 and 11:00 a.m. The leaf epidermis was carefully peeled off using a blade and tweezers and mounted on a slide. Pollen samples from the upper, middle, and lower tassel regions were collected, placed on slides, and stained with KI‐I_2_. Images were captured using an Olympus BX63 microscope and measured with Olympus CellSense Dimension 1.18 software.

### Photosynthetic and chlorophyll content

On a clear, cloudless morning between 9:00 and 11:00 a.m., photosynthetic parameters of the middle section of the first functional leaf from five plants were measured using an LI‐6400XT (LI‐COR, Lincoln, NE, USA). Test conditions included an airflow rate of 500 mmol s^−1^, irradiance of 1000 μmol m^−2^ s^−1^, leaf temperature of 24–26°C, relative humidity of 65–70%, and CO_2_ concentration of 400 μmol mol^−1^. Five measurements were taken per sample. Chlorophyll leaching assays were conducted on the first functional leaf by incubating 0.2 g of fresh leaf tissue on ice for 30 min, then immersing it in 20 ml of 80% ethanol at 25°C in darkness. Absorbance was measured at 647 and 664 nm using a spectrophotometer (Liu, Bourgault, et al., 2021), with at least three replicates per sample.

### Transcriptome analysis

#### RNA extraction and transcriptome sequencing

On the 105 d after transplanting, during vigorous vegetative growth, a 5 cm section (vein removed) from the middle part of the first functional leaf at the stem tip was sampled. Four biological replicates per plant line (plus one spare) were immediately frozen in liquid nitrogen and stored at −80°C for later analysis. Total RNA was extracted using the TRIzol reagent kit (Invitrogen, Carlsbad, CA, USA) according to the manufacturer's protocol, followed by treatment with DNase I (Thermo Scientific, USA). Agarose gel (1%) was used to detect RNA degradation and contamination. RNA purity was checked using NanoPhotometer® spectrophotometer (IMPLEN, CA, USA). RNA integrity was assessed using the Fragment Analyzer 5400 (Agilent Technologies, CA, USA). In total, 21 RNA‐seq libraries were constructed using an NEBNext® Ultra™ RNA Library Prep Kit for Illumina® (NEB, USA) following manufacturer's recommendations, index codes were added to attribute sequences to each sample, and 150 bp paired‐end reads were produced using the Illumina NovaSeq 6000 platform. Clean data were separated from the raw data by removing reads containing adapter, ploy‐N, and low‐quality reads. Meanwhile, Q20, Q30, GC‐content, and sequence duplication level of the clean data were also calculated. On the first functional leaf of vegetative growth phase (105 days after sowing) from 9475, MTP74, MTP94, MTP56, MTP67, MTP57, and MTP66, generating transcriptome atlases from approximately 270 Gb of clean data from 21 samples with three biological replicates (Table S9).

#### Quantification of gene expression level and identification of DEGs

Due to the lack of *Zea*–*Tripsacum* allopolyploid reference genomes, we used the previously reported PacBio full‐length transcriptome of allohexaploid trihybrid MTP74 as reference (Li et al., 2023). Illumina clean reads were mapped to the MTP74 transcriptome using Bowtie2. Batch effect were removed with the combat function in the R software SVA package (Müller et al., 2016). Gene expression level was normalized by fragments per kilobase per million mapped reads (FPKM) method using RSEM (Li & Dewey, 2011). Biological replicates were assessed via principal component analysis (PCA) in R (v4.1.1). Differentially expressed genes (DEGs) were identified using DESeq2 (Love et al., 2014), with *P*‐values adjusted by the Benjamini–Hochberg method to control the false discovery rate (FDR). Genes with FDR <0.05 and |log_2_ (Fold Change)| ≥ 1 were considered DEGs. Expression level dominance (ELD) and transgressive expression in F_1_ trihybrids versus parents were analyzed using Short Time‐series Expression Miner (STEM) software (Ernst & Bar‐Joseph, 2006).

#### WGCNA *analysis and gene function annotation*

Co‐expression networks were generated using WGCNA (v1.47) package in R software (v 4.1.1) (Langfelder & Horvath, 2008). Gene expression values were imported into WGCNA to construct co‐expression modules using the automatic network construction function blockwiseModules with default settings, except that the power is 12, TOMType is unsigned, mergeCutHeight is 0.9, minModuleSize is 20. Intramodular connectivity (K.in) and module correlation degree (MM) of each gene was calculated by R package of WGCNA and genes with high connectivity tended to be hub genes which might have important functions. Correlation analysis was performed using module eigengene with data for specific traits. Pearson correlation between each gene and trait data under the module is also calculated for the most relevant module (positive correlation and negative correlation) corresponding to each phenotype data, and gene significance value (GS) is obtained. Then, networks were visualized using Cytoscape_3.9.1 (Shannon et al., 2003). Gene Ontology (GO) enrichment and KEGG pathway enrichment analysis of functional genes were performed using the GOseq R packages (Young et al., 2010) and KOBAS software (Mao et al., 2005), The terms were considered to be significantly enriched if FDR <0.05.

### qRT‐PCR analysis

To validate the expression levels of genes within the modules significantly associated with biomass heterosis at the population level, we selected 10 genes from the modules that were significantly positively correlated with biomass heterosis, and compared with 10 genes selected from three modules that were significantly negatively correlated. In the pop79 and pop99, we selected 10 heterotic plant lines and compared with 11 disadvantageous plant lines. At the same time, we included diploid maize inbred lines Mo17, B73, and their hybrid Mo17 × B73 as extra reference. 105‐day‐old leaves were collected. Similarly, we also conducted expression level validation for the 205 dose‐related DEGs in different sample groups. RNA from different lines' leaves were extracted using the HiPure Plant RNA Mini Kit (Magen, Guangzhou, China) kit. Total RNAs were reverse transcribed using RevertAid First Strand cDNA Synthesis Kit (TaKaRa, Beijing, China) with random primers or oligo(dT)12–18 primers. RT‐qPCR analysis was conducted using SYBR® Premix Ex Taq™ II (TaKaRa, Beijing, China) on an ABI 7500 real‐time detection system. For each type, three independent biological replicates were analyzed, each with four technical replicates. The *ZmGAPDH* genes were used as reference controls, and the relative expression levels were calculated using the 2^−▵▵Ct^ method (Livak & Schmittgen, 2001).

## Author Contributions

QT, TR, XN, and YL designed and conceived experiments and managed the project. YL, XY, ZW, QZ, YZ, JL, SD, YZ, MC, XL, RH, HL, and JH participated in material collection and sample processing. YL performed the bioinformatics analyses. YL, XY, ZW, and QZ wrote the manuscript. YL, XY, and HL edited the manuscript.

## Conflict of Interest

The authors declare no competing interests.

## Supporting information


**Figure S1** MPH for each trait in pop79 population and pop99 population, respectively.
**Figure S2**. Analysis of panicle traits heterosis of representative materials from two populations.
**Figure S3**. Analysis of cell and stomatal size heterosis in representative materials of two populations.
**Figure S4**. Analysis of photosynthetic parameters heterosis of representative materials of two populations.
**Figure S5**. Pearson correlation analysis between important traits.
**Figure S6**. Effects of Z. perennis genome dosage on panicle traits of representative materials.
**Figure S7**. Effects of Z. perennis genome dosage on cell and stomatal size of representative materials.
**Figure S8**. Effects of Z. perennis genome dosage on photosynthesis of representative materials.
**Figure S9**. Identify of gene expression levels of different materials.
**Figure S10**. Heatmap of correlation of gene expression between 21 samples.
**Figure S11**. DEGs triggered by hybridity and their functional annotation in allopolyploids.
**Figure S12**. DEGs triggered by hybridity and their KEGG pathway in allopolyploids.
**Figure S13**. Analysis of expression pattern and KEGG pathway of polyploid hybrid F_1_s in perennial forage maize.
**Figure S14**. GO annotation of non‐additively expressed gene sets from heterosis and hybrid weakness materials.
**Figure S15**. KEGG enrichment in four modules significantly correlated with biomass.
**Figure S16**. Heatmap comparison of the DEGs for heterosis F1s (MTP56 and MTP67) and their female parents in protein quality control system, glutathione metabolism, spliceosome, energy and glucose metabolism, and metabolic pathway.
**Figure S17**. Co‐expression network of blue and brown module genes.
**Figure S18**. Validation of gene expression in negative correlation module at population level.
**Figure S19**. Expression of 205 dosage effect genes in representative materials of two populations.
**Figure S20**. Detection of pollen fertility of the pop79 sing *I*_2_‐KI staining.
**Figure S21**. Detection of pollen fertility of the pop99 sing *I*_2_‐KI staining.
**Figure S22**. Comparison of pollen fertility between pop79 and pop99.
**Figure S23**. Field scenes of the perennial forage maize.
**Table S1**. Karyotype summary of all pop79 population.
**Table S2**. Karyotype summary of all pop99 population.
**Table S3**. Phenotypic characteristics of MTP74 and MTP94 after allopolyploidization.
**Table S4**. Phenotypic characteristics of the pop79 population.
**Table S5**. Heterosis of phenotypic traits in pop79 population.
**Table S6**. Phenotypic characteristics of the pop99 population.
**Table S7**. Heterosis of phenotypic traits in pop99 population.
**Table S8**. Comparison of traits between pop79 and pop99 populations.
**Table S9**. Summary of the RNA‐seq data.
**Table S10**. Identify of gene expression levels of different materials.
**Table S11**. KEGG pathway of core DEGs in heterosis combination MTP74 × 9475 → MTP56.
**Table S12**. KEGG pathway of core DEGs in heterosis combination MTP94 × 9475 → MTP67.
**Table S13**. KEGG pathway of core DEGs shared by two heterosis combination.
**Table S14**. KEGG pathway of core DEGs in weakness combination MTP74 × 9475 → MTP57.
**Table S15**. KEGG pathway of core DEGs in weakness combination MTP94 × 9475 → MTP66.
**Table S16**. KEGG pathway of core DEGs shared by two weakness combinations.
**Table S17**. Hub genes in four modules significantly associated with fresh‐weight.
**Table S18**. Fresh yield of perennial forage maize variety in different ecological zones (t·ha^−1^).
**Table S19**. Hay yield of perennial forage maize variety in different ecological zones (t·ha^−1^).
**Table S20**. Mean hay and fresh yield of perennial forage maize variety in different ecological zones (t·ha^−1^).

## Data Availability

Supporting Information is available from the Wiley Online Library or from the author. RNA Sequencing data of can be found in Beijing Institute of Genomics Data Center (http://bigd.big.ac.cn) under the accession number PRJCA020360.

## References

[tpj71242-bib-0001] Banouh, M. , Armisen, D. , Bouguennec, A. , Huneau, C. , Sow, M.D. , Pont, C. et al. (2023) Low impact of polyploidization on the transcriptome of synthetic allohexaploid wheat. BMC Genomics, 24, 255. 10.1186/s12864-023-09324-2 PMC10173476 37170217

[tpj71242-bib-0002] Bastiaanse, H. , Zinkgraf, M. , Canning, C. , Tsai, H. , Lieberman, M. , Comai, L. et al. (2019) A comprehensive genomic scan reveals gene dosage balance impacts on quantitative traits in *Populus* trees. Proceedings of the National Academy of Sciences of the United States of America, 116, 13690–13699. 10.1073/pnas.1903229116 PMC6613180 31213538

[tpj71242-bib-0003] Bastiaanse, H.L.S. , Henry, I.M. , Tsai, H. , Lieberman, M. , Canning, C. , Comai, L. et al. (2021) A systems genetics approach to deciphering the effect of dosage variation on leaf morphology in *Populus* . The Plant Cell, 33, 940–960. 10.1093/plcell/koaa016 PMC8226299 33793772

[tpj71242-bib-0004] Batte, M. , Nyine, M. , Uwimana, B. , Swennen, R. , Akech, V. , Brown, A. et al. (2020) Significant progressive heterobeltiosis in banana crossbreeding. BMC Plant Biology, 2, 489. 10.1186/s12870-020-02667-y PMC7590486 33109087

[tpj71242-bib-0005] Bayne, B.L. (2004) Phenotypic flexibility and physiological tradeoffs in the feeding and growth of marine bivalve molluscs. Integrative and Comparative Biology, 44, 425–432. 10.1093/icb/44.6.425 21676728

[tpj71242-bib-0006] Benoit, M. , Jenike, K.M. , Satterlee, J.W. , Ramakrishnan, S. , Gentile, I. , Hendelman, A. et al. (2025) Solanum pan‐genetics reveals paralogues as contingencies in crop engineering. Nature, 640, 135–145. 10.1038/s41586-025-08619-6 PMC11964936 40044854

[tpj71242-bib-0007] Bingham, E.T. , Groose, R.W. , Woodfield, D.R. & Kidwell, K.K. (1994) Complementary gene interactions in *alfalfa* are greater in autotetraploids than diploids. Crop Science, 34, 823–829.

[tpj71242-bib-0009] Birchler, J.A. & Veitia, R.A. (2007) The gene balance hypothesis: from classical genetics to modern genomics. The Plant Cell, 19, 395–402. 10.1105/tpc.106.049338 PMC1867330 17293565

[tpj71242-bib-0010] Birchler, J.A. & Veitia, R.A. (2012) Gene balance hypothesis: connecting issues of dosage sensitivity across biological disciplines. Proceedings of the National Academy of Sciences of the United States of America, 109, 14746–14753. 10.1073/pnas.1207726109 PMC3443177 22908297

[tpj71242-bib-0011] Birchler, J.A. , Yao, H. , Chudalayandi, S. , Vaiman, D. & Veitia, R.A. (2010) Heterosis. The Plant Cell, 22, 2105–2112. 10.1105/tpc.110.076133 PMC2929104 20622146

[tpj71242-bib-0012] Bird, K.A. , Pires, J.C. , VanBuren, R. , Xiong, Z. & Edger, P.P. (2023) Dosage‐sensitivity shapes how genes transcriptionally respond to allopolyploidy and homoeologous exchange in resynthesized *Brassica napus* . Genetics, 225, iyad114. 10.1093/genetics/iyad114 PMC10471226 37338008

[tpj71242-bib-0013] Blanc, G. & Wolfe, K.H. (2004) Functional divergence of duplicated genes formed by polyploidy during Arabidopsis evolution. The Plant Cell, 16, 1679–1691. 10.1105/tpc.021410 PMC514153 15208398

[tpj71242-bib-0014] Brownfield, L. & Kohler, C. (2011) Unreduced gamete formation in plants: mechanisms and prospects. Journal of Experimental Botany, 62, 1659–1668. 10.1093/jxb/erq371 21109579

[tpj71242-bib-0015] Bruce, A.B. (1910) The mendelian theory of heredity and the augmentation of vigor. Science, 32, 627–628. 10.1126/science.32.827.627-a 17816706

[tpj71242-bib-0016] Buggs, R.J. , Zhang, L. , Miles, N. , Tate, J.A. , Gao, L. , Wei, W. et al. (2011) Transcriptomic shock generates evolutionary novelty in a newly formed, natural allopolyploid plant. Current Biology, 21, 551–556. 10.1016/j.cub.2011.02.016 21419627

[tpj71242-bib-0017] Burson, B.L. , Voigt, P.W. , Sherman, R.A. & Dewald, C.L. (1990) Apomixis and sexuality in eastern gamagrass. Crop Science, 30, 86–89.

[tpj71242-bib-0018] Chase, S.S. (1980) Studies of monoploid, diploid and tetraploids of maize in relation to heterosis and inbreeding depression. In: Proceedings of the Argentine Society of Genetics.

[tpj71242-bib-0019] Chelaifa, H. , Mahe, F. & Ainouche, M. (2010) Transcriptome divergence between the hexaploid salt‐marsh sister species *Spartina maritima* and *Spartina alterniflora* (Poaceae). Molecular Ecology, 19, 2050–2063. 10.1111/j.1365-294X.2010.04637.x 20550634

[tpj71242-bib-0020] Chelaifa, H. , Monnier, A. & Ainouche, M. (2010) Transcriptomic changes following recent natural hybridization and allopolyploidy in the salt marsh species *Spartina* × *townsendii* and *Spartina anglica* (Poaceae). New Phytologist, 186, 161–174. 10.1111/j.1469-8137.2010.03179.x 20149114

[tpj71242-bib-0021] Chen, Z.J. (2007) Genetic and epigenetic mechanisms for gene expression and phenotypic variation in plant polyploids. Annual Review of Plant Biology, 58, 377–406. 10.1146/annurev.arplant.58.032806.103835 PMC1949485 17280525

[tpj71242-bib-0022] Chen, Z.J. (2010) Molecular mechanisms of polyploidy and hybrid vigor. Trends in Plant Science, 15, 57–71. 10.1016/j.tplants.2009.12.003 PMC2821985 20080432

[tpj71242-bib-0023] Chester, M. , Gallagher, J.P. , Symonds, V.V. , Cruz da Silva, A.V. , Mavrodiev, E.V. , Leitch, A.R. et al. (2012) Extensive chromosomal variation in a recently formed natural allopolyploid species, *Tragopogon miscellus* (Asteraceae). Proceedings of the National Academy of Sciences of the United States of America, 109, 1176–1181. 10.1073/pnas.1112041109 PMC3268322 22228301

[tpj71242-bib-0024] Coate, J.E. , Schlueter, J.A. , Whaley, A.M. & Doyle, J.J. (2011) Comparative evolution of photosynthetic genes in response to polyploid and nonpolyploid duplication. Plant Physiology, 155, 2081–2095. 10.1104/pp.110.169599 PMC3091097 21289102

[tpj71242-bib-0025] Coate, J.E. , Song, M.J. , Bombarely, A. & Doyle, J.J. (2016) Expression‐level support for gene dosage sensitivity in three *glycine* subgenus *glycine* polyploids and their diploid progenitors. New Phytologist, 212, 1083–1093. 10.1111/nph.14090 27418296

[tpj71242-bib-0026] Comai, L. (2005) The advantages and disadvantages of being polyploid. Nature Reviews Genetics, 6, 836–846. 10.1038/nrg1711 16304599

[tpj71242-bib-0027] Crow, J.F. (1948) Alternative hypothesis of hybrid vigor. Genetics, 33, 477–487. 10.1093/genetics/33.5.477 PMC1209419 17247292

[tpj71242-bib-0028] Darwin, C.R. (1876) The effects of cross‐ and self‐fertilization in the vegetable kingdom. London: John Murry.

[tpj71242-bib-0029] Doyle, J.J. , Flagel, L.E. , Paterson, A.H. , Rapp, R.A. , Soltis, D.E. , Soltis, P.S. et al. (2008) Evolutionary genetics of genome merger and doubling in plants. Annual Review of Genetics, 42, 443–461. 10.1146/annurev.genet.42.110807.091524 18983261

[tpj71242-bib-0030] Duvick, D.N. (1992) Genetic contributions to advances in yield of U.S. maize. Maydica, 37, 69–79.

[tpj71242-bib-0031] Duvick, D.N. (1999) Heterosis: Feeding People and Protecting Natural Resources. In: Coors, J.G. & Pandey, S. (Eds.) Genetics and Exploitation of Heterosis in Crops. Madison: American Society of America, pp. 19–29.

[tpj71242-bib-0032] East, E.M. (1936) Heterosis. Genetics, 21, 375–397. 10.1093/genetics/21.4.375 PMC1208682 17246801

[tpj71242-bib-0033] Ernst, J. & Bar‐Joseph, Z. (2006) STEM: a tool for the analysis of short time series gene expression data. BMC Bioinformatics, 7, 191. 10.1186/1471-2105-7-191 PMC1456994 16597342

[tpj71242-bib-0034] Fort, A. , Ryder, P. , McKeown, P.C. , Wijnen, C. , Aarts, M.G. , Sulpice, R. et al. (2016) Disaggregating polyploidy, parental genome dosage and hybridity contributions to heterosis in *Arabidopsis thaliana* . New Phytologist, 209, 590–599. 10.1111/nph.13650 26395035

[tpj71242-bib-0035] Fox, D.T. , Soltis, D.E. , Soltis, P.S. , Ashman, T.L. & Van de Peer, Y. (2020) Polyploidy: a biological force from cells to ecosystems. Trends in Cell Biology, 30, 688–694. 10.1016/j.tcb.2020.06.006 PMC7484144 32646579

[tpj71242-bib-0036] Freeling, M. (2017) Picking up the ball at the K/Pg boundary: the distribution of ancient polyploidies in the plant phylogenetic tree as a spandrel of asexuality with occasional sex. The Plant Cell, 29, 202–206. 10.1105/tpc.16.00836 PMC5354197 28213362

[tpj71242-bib-0037] Fu, D. , Xiao, M. , Hayward, A. , Fu, Y. , Liu, G. , Jiang, G. et al. (2014) Utilization of crop heterosis: a review. Euphytica, 197, 161–173.

[tpj71242-bib-0039] Ginn, B.R. (2010) The implications of gene heterozygosity for protein folding and protein turnover. Journal of Theoretical Biology, 265, 554–564. 10.1016/j.jtbi.2010.05.023 20493885

[tpj71242-bib-0040] Ginn, B.R. (2017) The thermodynamics of protein aggregation reactions may underpin the enhanced metabolic efficiency associated with heterosis, some balancing selection, and the evolution of ploidy levels. Progress in Biophysics & Molecular biology, 126, 1–21. 10.1016/j.pbiomolbio.2017.01.005 28185903

[tpj71242-bib-0042] Goff, S.A. (2011) A unifying theory for general multigenic heterosis: energy efficiency, protein metabolism, and implications for molecular breeding. New Phytologist, 189, 923–937. 10.1111/j.1469-8137.2010.03574.x 21166808

[tpj71242-bib-0043] Goff, S.A. & Zhang, Q. (2013) Heterosis in elite hybrid rice: speculation on the genetic and biochemical mechanisms. Current Opinion in Plant Biology, 16, 221–227. 10.1016/j.pbi.2013.03.009 23587937

[tpj71242-bib-0044] Grimanelli, D. , Leblanc, O. , Espinosa, E. , Perotti, E. , González de León, D. & Savidan, Y. (1998) Mapping diplosporous apomixis in tetraploid *Tripsacum*: one gene or several genes? Heredity, 80, 33–39. 10.1046/j.1365-2540.1998.00263.x 9474774

[tpj71242-bib-0045] Groose, R.W. , Talbert, L.E. , Kojis, W.P. & Bingham, E.T. (1989) Progressive heterosis in autotetraploid *alfalfa*: studies using two types of inbreds. Crop Science, 29, 1173–1177.

[tpj71242-bib-0046] Guo, M. , Davis, D. & Birchler, J.A. (1996) Dosage effects on gene expression in a maize ploidy series. Genetics, 142, 1349–1355. 10.1093/genetics/142.4.1349 PMC1207130 8846910

[tpj71242-bib-0047] Hao, M. , Li, A. , Shi, T. , Luo, J. , Zhang, L. , Zhang, X. et al. (2017) The abundance of homoeologue transcripts is disrupted by hybridization and is partially restored by genome doubling in synthetic hexaploid wheat. BMC Genomics, 18, 149. 10.1186/s12864-017-3558-0 PMC5303294 28187716

[tpj71242-bib-0048] Hawkins, A.J.S. , Bayne, B.L. , Day, A.J. & Denton, E.J. (1986) Protein turnover, physiological energetics and heterozygosity in the blue mussel, *Mytilus edulis*: the basis of variable age‐specific growth. Proceedings of the Royal Society of London, Series B: Biological Sciences, 229, 161–176.

[tpj71242-bib-0049] He, F. , Wang, W. , Rutter, W.B. , Jordan, K.W. , Ren, J. , Taagen, E. et al. (2022) Genomic variants affecting homoeologous gene expression dosage contribute to agronomic trait variation in allopolyploid wheat. Nature Communications, 13, 826. 10.1038/s41467-022-28453-y PMC8837796 35149708

[tpj71242-bib-0050] He, R.Y. , Yang, T. , Zheng, J.J. , Pan, Z.Y. , Chen, Y. , Zhou, Y. et al. (2023) QTL mapping and a transcriptome integrative analysis uncover the candidate genes that control the cold tolerance of maize introgression lines at the seedling stage. International Journal of Molecular Sciences, 24, 2629. 10.3390/ijms24032629 PMC9917090 36768951

[tpj71242-bib-0051] He, R.Y. , Zheng, J.J. , Chen, Y. , Pan, Z.Y. , Yang, T. , Zhou, Y. et al. (2023) QTL‐seq and transcriptomic integrative analyses reveal two positively regulated genes that control the low‐temperature germination ability of MTP‐maize introgression lines. Theoretical and Applied Genetics, 136, 116. 10.1007/s00122-023-04362-6 37093290

[tpj71242-bib-0052] Hedgecock, D. , McGoldrick, D.J. , Manahan, D.T. , Vavra, J. , Appelmans, N. & Bayne, B. (1996) Quantitative and molecular genetic analyses of heterosis in bivalve molluscs. Journal of Experimental Marine Biology and Ecology, 203, 49–59.

[tpj71242-bib-0053] Hegarty, M.J. , Barker, G.L. , Wilson, I.D. , Abbott, R.J. , Edwards, K.J. & Hiscock, S.J. (2006) Transcriptome shock after interspecific hybridization in senecio is ameliorated by genome duplication. Current Biology, 16, 1652–1659. 10.1016/j.cub.2006.06.071 16920628

[tpj71242-bib-0054] Henry, I.M. , Zinkgraf, M.S. , Groover, A.T. & Comai, L. (2015) A system for dosage‐based functional genomics in *poplar* . The Plant Cell, 27, 2370–2383. 10.1105/tpc.15.00349 PMC4815095 26320226

[tpj71242-bib-0055] Hochholdinger, F. & Hoecker, N. (2007) Towards the molecular basis of heterosis. Trends in Plant Science, 12, 427–432. 10.1016/j.tplants.2007.08.005 17720610

[tpj71242-bib-0056] Hochholdinger, F. & Yu, P. (2025) Molecular concepts to explain heterosis in crops. Trends in Plant Science, 30, 95–104. 10.1016/j.tplants.2024.07.018 39191625

[tpj71242-bib-0057] Hou, J. , Shi, X. , Chen, C. , Islam, M.S. , Johnson, A.F. , Kanno, T. et al. (2018) Global impacts of chromosomal imbalance on gene expression in Arabidopsis and other taxa. Proceedings of the National Academy of Sciences of the United States of America, 115, E11321–E11330. 10.1073/pnas.1807796115 PMC6275517 30429332

[tpj71242-bib-0058] Iqbal, M.Z. , Cheng, M. , Su, Y. , Li, Y. , Jiang, W. , Li, H. et al. (2019) Allopolyploidization facilitates gene flow and speciation among corn, *Zea perennis* and *Tripsacum dactyloides* . Planta, 249, 1949–1962. 10.1007/s00425-019-03136-z 30895446

[tpj71242-bib-0059] Iqbal, M.Z. , Wen, X. , Xu, L. , Zhao, Y. , Li, J. , Jiang, W. et al. (2023) Multispecies polyploidization, chromosome shuffling, and genome extraction in *Zea*/*Tripsacum* hybrids. Genetics, 223, iyad029. 10.1093/genetics/iyad029 36807971

[tpj71242-bib-0060] Jansky, S. (2009) Chapter 2–Breeding, genetics, and cultivar development. San Diego: Academic Press, pp. 27–62.

[tpj71242-bib-0061] Jones, D.F. (1917) Dominance of linked factors as a means of accounting for heterosis. Genetics, 2, 466–479. 10.1093/genetics/2.5.466 PMC1193729 17245892

[tpj71242-bib-0062] June, V. , Xu, D. , Papoulas, O. , Boutz, D. , Marcotte, E.M. & Chen, Z.J. (2023) Protein nonadditive expression and solubility contribute to heterosis in Arabidopsis hybrids and allotetraploids. Frontiers in Plant Science, 14, 1252564. 10.3389/fpls.2023.1252564 PMC10538547 37780492

[tpj71242-bib-0063] Kindiger, B. , Sokolov, V. & Dewald, C. (1996) A comparison of apomictic reproduction in eastern gamagrass (*Tripsacum dactyloides* (L.) L.) and maize‐*Tripsacum* hybrids. Genetica, 97, 103–110.

[tpj71242-bib-0064] Kindiger, B. , Sokolov, V. & Khatypova, I.V. (1996) Evaluation of apomictic reproduction in a set of 39 chromosome maize‐*Tripsacum* backcross hybrids. Crop Science, 36, 1108–1113.

[tpj71242-bib-0065] Ko, D.K. , Rohozinski, D. , Song, Q. , Taylor, S.H. , Juenger, T.E. , Harmon, F.G. et al. (2016) Temporal shift of circadian‐mediated gene expression and carbon fixation contributes to biomass heterosis in maize hybrids. PLoS Genetics, 12, e1006197. 10.1371/journal.pgen.1006197 PMC4965137 27467757

[tpj71242-bib-0067] Kristensen, T.N. , Dahlgaard, J. & Loeschcke, V. (2002) Inbreeding affects Hsp70 expression in two species of *drosophila* even at benign temperatures. Evolutionary Ecology Research, 4, 1209–1216.

[tpj71242-bib-0068] Langfelder, P. & Horvath, S. (2008) WGCNA: an R package for weighted correlation network analysis. BMC Bioinformatics, 9, 559. 10.1186/1471-2105-9-559 PMC2631488 19114008

[tpj71242-bib-0069] Leitch, A.R. & Leitch, I.J. (2008) Genomic plasticity and the diversity of polyploid plants. Science, 320, 481–483. 10.1126/science.1153585 18436776

[tpj71242-bib-0070] Li, A. , Liu, D. , Wu, J. , Zhao, X. , Hao, M. , Geng, S. et al. (2014) mRNA and small RNA transcriptomes reveal insights into dynamic homoeolog regulation of allopolyploid heterosis in nascent hexaploid wheat. The Plant Cell, 26, 1878–1900. 10.1105/tpc.114.124388 PMC4079356 24838975

[tpj71242-bib-0071] Li, B. & Dewey, C.N. (2011) RSEM: accurate transcript quantification from RNA‐Seq data with or without a reference genome. BMC Bioinformatics, 12, 323. 10.1186/1471-2105-12-323 PMC3163565 21816040

[tpj71242-bib-0072] Li, P. , Su, T. , Zhang, D. , Wang, W. , Xin, X. , Yu, Y. et al. (2021) Genome‐wide analysis of changes in miRNA and target gene expression reveals key roles in heterosis for Chinese cabbage biomass. Horticulture Research, 8, 39. 10.1038/s41438-021-00474-6 PMC7917107 33642594

[tpj71242-bib-0074] Li, X. , Wang, X. , Ma, Q. , Zhong, Y. , Zhang, Y. , Zhang, P. et al. (2023) Integrated single‐molecule real‐time sequencing and RNA sequencing reveal the molecular mechanisms of salt tolerance in a novel synthesized polyploid genetic bridge between maize and its wild relatives. BMC Genomics, 24, 55. 10.1186/s12864-023-09148-0 PMC9887930 36717785

[tpj71242-bib-0075] Li, Y. , Lu, Y. , Zhou, Y. , Wei, X. , Peng, Y. , Dai, Y. et al. (2021) Diurnal transcriptomics analysis reveals the regulatory role of the circadian rhythm in super‐hybrid rice LY2186. Genomics, 113, 1281–1290. 10.1016/j.ygeno.2020.12.046 33705889

[tpj71242-bib-0076] Li, Y. , Yan, X. , Cheng, M. , Wu, Z. , Zhang, Q. , Duan, S. et al. (2024) Genome dosage alteration caused by chromosome pyramiding and shuffling effects on karyotypic heterogeneity, reproductive diversity, and phenotypic variation in *Zea*‐*Tripsacum* allopolyploids. Theoretical and Applied Genetics, 137, 28. 10.1007/s00122-023-04540-6 38252297

[tpj71242-bib-0077] Li, Y. , Yan, X. , Wu, Z. , Yang, C. , Li, X. , He, R. et al. (2019) Forage maize type and growth stage effects on biomass yield and silage quality. Acta Prataculturae Sinica, 28, 82–91.

[tpj71242-bib-0078] Li, Z. , Zhu, A. , Song, Q. , Chen, H.Y. , Harmon, F.G. & Chen, Z.J. (2020) Temporal regulation of the metabolome and proteome in photosynthetic and photorespiratory pathways contributes to maize heterosis. The Plant Cell, 32, 3706–3722. 10.1105/tpc.20.00320 PMC7721322 33004616

[tpj71242-bib-0079] Liu, W. , He, G. & Deng, X.W. (2021) Biological pathway expression complementation contributes to biomass heterosis in Arabidopsis. Proceedings of the National Academy of Sciences of the United States of America, 118, e2023278118. 10.1073/pnas.2023278118 PMC8072403 33846256

[tpj71242-bib-0080] Liu, X. , Bourgault, R. , Galli, M. , Strable, J. , Chen, Z. , Feng, F. et al. (2021) The FUSED LEAVES1‐ADHERENT1 regulatory module is required for maize cuticle development and organ separation. New Phytologist, 229, 388–402. 10.1111/nph.16837 PMC7754373 32738820

[tpj71242-bib-0081] Livak, K. & Schmittgen, T. (2001) Analysis of relative gene expression data using real‐time quantitative PCR and the 2(‐Delta Delta C(T)) method. Methods (San Diego, Calif.), 25, 402–408. 10.1006/meth.2001.1262 11846609

[tpj71242-bib-0082] Love, M.I. , Huber, W. & Anders, S. (2014) Moderated estimation of fold change and dispersion for RNA‐seq data with DESeq2. Genome Biology, 15, 550. 10.1186/s13059-014-0550-8 PMC4302049 25516281

[tpj71242-bib-0083] Mackay, I.J. , Cockram, J. , Howell, P. & Powell, W. (2021) Understanding the classics: the unifying concepts of transgressive segregation, inbreeding depression and heterosis and their central relevance for crop breeding. Plant Biotechnology Journal, 19, 26–34. 10.1111/pbi.13481 PMC7769232 32996672

[tpj71242-bib-0084] Madani, H. , Escrich, A. , Hosseini, B. , Sanchez‐Muñoz, R. , Khojasteh, A. & Palazon, J. (2021) Effect of polyploidy induction on natural metabolite production in medicinal plants. Biomolecules, 11, 899. 10.3390/biom11060899 PMC8234191 34204200

[tpj71242-bib-0085] Maere, S. , De Bodt, S. , Raes, J. , Casneuf, T. , Van Montagu, M. & Kuiper, M. (2005) Modeling gene and genome duplications in eukaryotes. Proceedings of the National Academy of Sciences of the United States of America, 102, 5454–5459. 10.1073/pnas.0501102102 PMC556253 15800040

[tpj71242-bib-0086] Mao, X. , Cai, T. , Olyarchuk, J.G. & Wei, L. (2005) Automated genome annotation and pathway identification using the KEGG Orthology (KO) as a controlled vocabulary. Bioinformatics, 21, 3787–3793. 10.1093/bioinformatics/bti430 15817693

[tpj71242-bib-0087] Miller, M. , Song, Q. , Shi, X. , Juenger, T.E. & Chen, Z.J. (2015) Natural variation in timing of stress‐responsive gene expression predicts heterosis in intraspecific hybrids of Arabidopsis. Nature Communications, 6, 7453. 10.1038/ncomms8453 26154604

[tpj71242-bib-0088] Mok, D.W. & Peloquin, S.J. (1975) Breeding value of 2*n* pollen (diplandroids) in tetraploid × diploid crosses in potatoes. Theoretical and Applied Genetics, 46, 307–314. 10.1007/BF00281153 24420125

[tpj71242-bib-0089] Müller, C. , Schillert, A. , Röthemeier, C. , Trégouët, D.A. , Proust, C. , Binder, H. et al. (2016) Removing batch effects from longitudinal gene expression ‐ quantile normalization plus combat as best approach for microarray transcriptome data. PLoS One, 11, e0156594. 10.1371/journal.pone.0156594 PMC4896498 27272489

[tpj71242-bib-0090] Ni, Z. , Kim, E.D. , Ha, M. , Lackey, E. , Liu, J. , Zhang, Y. et al. (2009) Altered circadian rhythms regulate growth vigor in hybrids and allopolyploids. Nature, 457, 327–331. 10.1038/nature07523 PMC2679702 19029881

[tpj71242-bib-0091] Okubo, K. & Kaneko, K. (2022) Heterosis of fitness and phenotypic variance in the evolution of a diploid gene regulatory network. PNAS Nexus, 1, pgac097. 10.1093/pnasnexus/pgac097 PMC9896930 36741431

[tpj71242-bib-0092] Orr‐Weaver, T.L. (2015) When bigger is better: the role of polyploidy in organogenesis. Trends in Genetics, 31, 307–315. 10.1016/j.tig.2015.03.011 PMC4537166 25921783

[tpj71242-bib-0093] Otto, S.P. (2007) The evolutionary consequences of polyploidy. Cell, 131, 452–462. 10.1016/j.cell.2007.10.022 17981114

[tpj71242-bib-0094] Pedersen, K.S. , Kristensen, T.N. & Loeschcke, V. (2005) Effects of inbreeding and rate of inbreeding in *Drosophila* melanogaster‐Hsp70 expression and fitness. Journal of Evolutionary Biology, 18, 756–762. 10.1111/j.1420-9101.2005.00884.x 16033546

[tpj71242-bib-0095] Qin, J. , Mo, R. , Li, H. , Ni, Z. , Sun, Q. & Liu, Z. (2021) The transcriptional and splicing changes caused by hybridization can be globally recovered by genome doubling during allopolyploidization. Molecular Biology and Evolution, 38, 2513–2519. 10.1093/molbev/msab045 PMC8136492 33585937

[tpj71242-bib-0096] Roy, A.K. , Chakraborti, M. , Radhakrishna, A. , Dwivedi, K.K. , Srivastava, M.K. , Saxena, S. et al. (2022) Alien genome mobilization and fixation utilizing an apomixis mediated genome addition (AMGA) strategy in *Pennisetum* to improve domestication traits of *P. squamulatum* . Theoretical and Applied Genetics, 135, 2555–2575. 10.1007/s00122-022-04138-4 35726065

[tpj71242-bib-0097] Salmon, A. , Ainouche, M.L. & Wendel, J.F. (2005) Genetic and epigenetic consequences of recent hybridization and polyploidy in *Spartina* (Poaceae). Molecular Ecology, 14, 1163–1175. 10.1111/j.1365-294X.2005.02488.x 15773943

[tpj71242-bib-0098] Schnell, F.W. & Cockerham, C.C. (1992) Multiplicative vs. arbitrary gene action in heterosis. Genetics, 131, 461–469. 10.1093/genetics/131.2.461 PMC1205018 1644280

[tpj71242-bib-0099] Schoenfelder, K.P. & Fox, D.T. (2015) The expanding implications of polyploidy. The Journal of Cell Biology, 209, 485–491. 10.1083/jcb.201502016 PMC4442802 26008741

[tpj71242-bib-0100] Shahzad, K. , Zhang, X. , Guo, L. , Qi, T. , Tang, H. , Zhang, M. et al. (2020) Comparative transcriptome analysis of inbred lines and contrasting hybrids reveals overdominance mediate early biomass vigor in hybrid cotton. BMC Genomics, 21, 140. 10.1186/s12864-020-6561-9 PMC7011360 32041531

[tpj71242-bib-0101] Shannon, P. , Markiel, A. , Ozier, O. , Baliga, N.S. , Wang, J.T. , Ramage, D. et al. (2003) Cytoscape: a software environment for integrated models of biomolecular interaction networks. Genome Research, 13, 2498–2504. 10.1101/gr.1239303 PMC403769 14597658

[tpj71242-bib-0102] Shen, G. , Zhan, W. , Chen, H. & Xing, Y. (2014) Dominance and epistasis are the main contributors to heterosis for plant height in rice. Plant Science, 215‐216, 11–18. 10.1016/j.plantsci.2013.10.004 24388510

[tpj71242-bib-0104] Shi, X. , Yang, H. , Chen, C. , Hou, J. , Hanson, K.M. , Albert, P.S. et al. (2021) Genomic imbalance determines positive and negative modulation of gene expression in diploid maize. The Plant Cell, 33, 917–939. 10.1093/plcell/koab030 PMC8226301 33677584

[tpj71242-bib-0105] Shull, G.H. (1908) The composition of a field of maize. Journal of Heredity, 4, 296–301.

[tpj71242-bib-0106] Sinha, P. , Singh, V.K. , Saxena, R.K. , Kale, S.M. , Li, Y. , Garg, V. et al. (2020) Genome‐wide analysis of epigenetic and transcriptional changes associated with heterosis in pigeonpea. Plant Biotechnology Journal, 18, 1697–1710. 10.1111/pbi.13333 PMC7336283 31925873

[tpj71242-bib-0107] Song, L. , Shi, J.Y. , Duan, S.F. , Han, D.Y. , Li, K. , Zhang, R.P. et al. (2021) Improved redox homeostasis owing to the up‐regulation of one‐carbon metabolism and related pathways is crucial for yeast heterosis at high temperature. Genome Research, 31, 622–634. 10.1101/gr.262055.120 PMC8015850 33722936

[tpj71242-bib-0110] Sun, S. , Wang, B. , Li, C. , Xu, G. , Yang, J. , Hufford, M.B. et al. (2023) Unraveling prevalence and effects of deleterious mutations in maize elite lines across decades of modern breeding. Molecular Biology and Evolution, 40, msad170. 10.1093/molbev/msad170 PMC10414807 37494285

[tpj71242-bib-0111] Tian, M. , Nie, Q. , Li, Z. , Zhang, J. , Liu, Y. , Long, Y. et al. (2018) Transcriptomic analysis reveals overdominance playing a critical role in nicotine heterosis in *Nicotiana tabacum* L. BMC Plant Biology, 18, 48. 10.1186/s12870-018-1257-x PMC5863848 29566653

[tpj71242-bib-0112] Toda, E. & Okamoto, T. (2020) Polyspermy in angiosperms: its contribution to polyploid formation and speciation. Molecular Reproduction and Development, 87, 374–379. 10.1002/mrd.23295 31736192

[tpj71242-bib-0113] Torgeman, S. & Zamir, D. (2023) Epistatic QTLs for yield heterosis in tomato. Proceedings of the National Academy of Sciences of the United States of America, 120, e2205787119. 10.1073/pnas.2205787119 PMC10083602 36972451

[tpj71242-bib-0114] Troyer, A.F. & Wellin, E.J. (2009) Heterosis decreasing in hybrids: yield test inbreds. Crop Science, 49, 1969–1976.

[tpj71242-bib-0115] Van de Peer, Y. , Ashman, T.L. , Soltis, P.S. & Soltis, D.E. (2021) Polyploidy: an evolutionary and ecological force in stressful times. The Plant Cell, 33, 11–26. 10.1093/plcell/koaa015 PMC8136868 33751096

[tpj71242-bib-0116] Van de Peer, Y. , Mizrachi, E. & Marchal, K. (2017) The evolutionary significance of polyploidy. Nature Reviews Genetics, 18, 411–424. 10.1038/nrg.2017.26 28502977

[tpj71242-bib-0117] Veitia, R.A. & Birchler, J.A. (2022) Gene‐dosage issues: a recurrent theme in whole genome duplication events. Trends in Genetics, 38, 1–3. 10.1016/j.tig.2021.06.006 34215425

[tpj71242-bib-0118] Wang, B. , Hou, M. , Shi, J. , Ku, L. , Song, W. , Li, C. et al. (2023) *De novo* genome assembly and analyses of 12 founder inbred lines provide insights into maize heterosis. Nature Genetics, 55, 312–323. 10.1038/s41588-022-01283-w 36646891

[tpj71242-bib-0119] Wang, H. , Wang, R. , Chen, B. , Aslam, T. , Lian, T. , Xu, C. et al. (2026) Aneuploidy confers environmentally robust higher evolvability than euploidy in a synthetic allotetraploid wheat. Science Advances, 12, eaec8789. 10.1126/sciadv.aec8789 PMC13134582 42066068

[tpj71242-bib-0120] Wang, J. , Tian, L. , Lee, H.S. , Wei, N.E. , Jiang, H. , Watson, B. et al. (2006) Genomewide nonadditive gene regulation in Arabidopsis allotetraploids. Genetics, 172, 507–517. 10.1534/genetics.105.047894 PMC1456178 16172500

[tpj71242-bib-0121] Wang, Y. , Fuentes, R.R. , Van Rengs, W.M.J. , Effgen, S. , Zaidan, M.W.A.M. , Franzen, R. et al. (2024) Harnessing clonal gametes in hybrid crops to engineer polyploid genomes. Nature Genetics, 56, 1075–1079. 10.1038/s41588-024-01750-6 PMC11176054 38741016

[tpj71242-bib-0122] Washburn, J.D. & Birchler, J.A. (2014) Polyploids as a “model system” for the study of heterosis. Plant Reproduction, 27, 1–5. 10.1007/s00497-013-0237-4 24202960

[tpj71242-bib-0123] Washburn, J.D. , McElfresh, M.J. & Birchler, J.A. (2019) Progressive heterosis in genetically defined tetraploid maize. Journal of Genetics and Genomics, 46, 389–396. 10.1016/j.jgg.2019.02.010 31444136

[tpj71242-bib-0125] Yan, X. , Cheng, M. , Li, Y. , Wu, Z. , Li, Y. , Li, X. et al. (2020) Tripsazea, a novel trihybrid of *Zea mays*, *Tripsacum dactyloides*, and *Zea perennis* . G3: Genes, Genomes, Genetics, 10, 839–848. 10.1534/g3.119.400942 PMC7003090 31792004

[tpj71242-bib-0126] Yan, X. , Li, Y. , Wu, Z. , Li, Y. , Wen, X. , Li, X. et al. (2020) Analysis of the genitor origin of an intergeneric hybrid clone between *Zea* and *Tripsacum* for forage production by McGISH. Breeding Science, 70, 241–245. 10.1270/jsbbs.19107 PMC7272247 32523406

[tpj71242-bib-0127] Yang, H. , Shi, X. , Chen, C. , Hou, J. , Ji, T. , Cheng, J. et al. (2021) Predominantly inverse modulation of gene expression in genomically unbalanced disomic haploid maize. The Plant Cell, 33, 901–916. 10.1093/plcell/koab029 PMC8226288 33656551

[tpj71242-bib-0128] Yang, L. , Liu, P. , Wang, X. , Jia, A. , Ren, D. , Tang, Y. et al. (2021) A central circadian oscillator confers defense heterosis in hybrids without growth vigor costs. Nature Communications, 12, 2317. 10.1038/s41467-021-22268-z PMC8055661 33875651

[tpj71242-bib-0129] Yao, H. , Dogra Gray, A. , Auger, D.L. & Birchler, J.A. (2013) Genomic dosage effects on heterosis in triploid maize. Proceedings of the National Academy of Sciences of the United States of America, 110, 2665–2669. 10.1073/pnas.1221966110 PMC3574931 23359717

[tpj71242-bib-0130] Yoo, M.J. , Szadkowski, E. & Wendel, J. (2013) Homoeolog expression bias and expression level dominance in allopolyploid cotton. Heredity, 110, 171–180. 10.1038/hdy.2012.94 PMC3554454 23169565

[tpj71242-bib-0131] Young, M.D. , Wakefield, M.J. , Smyth, G.K. & Oshlack, A. (2010) Gene ontology analysis for RNA‐seq: accounting for selection bias. Genome Biology, 11, R14. 10.1186/gb-2010-11-2-r14 PMC2872874 20132535

[tpj71242-bib-0132] Yu, S.B. , Li, J.X. , Xu, C.G. , Tan, Y.F. , Gao, Y.J. , Li, X.H. et al. (1997) Importance of epistasis as the genetic basis of heterosis in an elite rice hybrid. Proceedings of the National Academy of Sciences of the United States of America, 94, 9226–9231. 10.1073/pnas.94.17.9226 PMC23127 11038567

[tpj71242-bib-0133] Yu, Z. , Cui, B. , Xiao, J. , Jiao, W. , Wang, H. , Wang, Z. et al. (2024) Dosage effect genes modulate grain development in synthesized *Triticum durum*‐*Haynaldia villosa* allohexaploid. Journal of Genetics and Genomics, 51, 1089–1100. 10.1016/j.jgg.2024.04.010 38670432

[tpj71242-bib-0134] Zhang, H. , Bian, Y. , Gou, X. , Zhu, B. , Xu, C. , Qi, B. et al. (2013) Persistent whole‐chromosome aneuploidy is generally associated with nascent allohexaploid wheat. Proceedings of the National Academy of Sciences of the United States of America, 110, 3447–3452. 10.1073/pnas.1300153110 PMC3587266 23401544

[tpj71242-bib-0135] Zhang, X. , Tang, C. , Jiang, B. , Zhang, R. , Li, M. , Wu, Y. et al. (2025) Refining polyploid breeding in sweet potato through allele dosage enhancement. Nature Plants, 11, 36–48. 10.1038/s41477-024-01873-y 39668213

[tpj71242-bib-0136] Zhao, K. , Dong, J. , Xu, J. , Bai, Y. , Yin, Y. , Long, C. et al. (2023) Downregulation of the expression of subgenomic chromosome A7 genes promotes plant height in resynthesized allopolyploid *Brassica napus* . Theoretical and Applied Genetics, 137, 11. 10.1007/s00122-023-04510-y 38110525

[tpj71242-bib-0137] Zhou, Y. , Li, Y. , Luo, L. , Zhang, D. , Wang, X. , Chen, Y. et al. (2025) Maize‐*Tripsacum*‐teosinte allopolyploid (MTP), a novel dwarf mutant inducer tool in maize. Plant Biotechnology Journal, 23, 112–127. 10.1111/pbi.14483 PMC11672742 39361445

[tpj71242-bib-0138] Zhu, A. , Wang, A. , Zhang, Y. , Dennis, E.S. , Peacock, W.J. & Greaves, A.I.K. (2020) Early establishment of photosynthesis and auxin biosynthesis plays a key role in early biomass heterosis in *Brassica napus* (canola) hybrids. Plant and Cell Physiology, 61, 1134–1143. 10.1093/pcp/pcaa038 32215572

