# Supplementary material for: Deciphering the contributions of hybridization and genome dosage to vegetative vigor in perennial forage maize
Source: Plant J. 2026 Nov 10;128(3):e71242. doi: 10.1111/tpj.71242 (PMC13749249; doi:10.1111/tpj.71242)
Supplement: Supplementary file 1 [file TPJ-128-0-s001.docx]

**Table S1. Karyotype summary of all pop79 population**

| Material | Parantal chromosomes | | | Total chromosomes | Translocation chromosomes | | | | | Total Translocations | Reproduction |
| --- | --- | --- | --- | --- | --- | --- | --- | --- | --- | --- | --- |
|  | M | T | P |  | MP | PM | TP | MT | PT |  |  |
| 7901 | 10 | 17 | 30 | 57 | 0 | 1 | 0 | 0 | 0 | 1 | n+n |
| 7902 | 12 | 16 | 27 | 55 | 0 | 0 | 0 | 0 | 0 | 0 | n+n |
| 7903 | 10 | 16 | 29 | 55 | 0 | 0 | 0 | 0 | 0 | 0 | n+n |
| 7904 | 11 | 16 | 29 | 56 | 0 | 0 | 0 | 0 | 0 | 0 | n+n |
| 7905 | 12 | 16 | 31 | 59 | 0 | 0 | 0 | 0 | 0 | 0 | n+n |
| 7906 | 11 | 16 | 30 | 57 | 1 | 1 | 0 | 0 | 0 | 2 | n+n |
| 7907 | 8 | 16 | 30 | 54 | 0 | 1 | 0 | 0 | 0 | 1 | n+n |
| 7908 | 10 | 17 | 30 | 57 | 0 | 0 | 0 | 0 | 0 | 0 | n+n |
| 7909 | 8 | 18 | 32 | 58 | 0 | 0 | 0 | 0 | 0 | 0 | n+n |
| 7910 | 13 | 15 | 26 | 54 | 0 | 0 | 0 | 0 | 0 | 0 | n+n |
| 7911 | 11 | 15 | 29 | 55 | 0 | 0 | 0 | 0 | 0 | 0 | n+n |
| 7912 | 12 | 15 | 27 | 54 | 0 | 0 | 0 | 0 | 0 | 0 | n+n |
| 7913 | 7 | 18 | 33 | 58 | 0 | 0 | 0 | 0 | 0 | 0 | n+n |
| 7915 | 20 | 33 | 36 | 89 | 0 | 0 | 0 | 0 | 0 | 0 | 2n+n |
| 7916 | 12 | 16 | 26 | 54 | 0 | 0 | 0 | 0 | 0 | 0 | n+n |
| 7917 | 10 | 16 | 30 | 56 | 0 | 0 | 0 | 0 | 0 | 0 | n+n |
| 7918 | 10 | 17 | 30 | 57 | 0 | 0 | 0 | 0 | 0 | 0 | n+n |
| 7920 | 18 | 33 | 31 | 82 | 0 | 0 | 0 | 0 | 0 | 0 | 2n+n |
| 7921 | 8 | 15 | 14 | 37 | 0 | 0 | 0 | 0 | 0 | 0 | n |
| 7924 | 12 | 17 | 28 | 57 | 0 | 0 | 0 | 0 | 0 | 0 | n+n |
| 7927 | 12 | 16 | 27 | 55 | 0 | 0 | 0 | 0 | 0 | 0 | n+n |
| 7928 | 10 | 16 | 32 | 58 | 0 | 0 | 0 | 0 | 0 | 0 | n+n |
| 7930 | 10 | 16 | 30 | 56 | 2 | 0 | 0 | 0 | 0 | 2 | n+n |
| 7932 | 10 | 15 | 30 | 55 | 0 | 0 | 0 | 0 | 0 | 0 | n+n |
| 7933 | 9 | 18 | 30 | 57 | 0 | 0 | 0 | 0 | 0 | 0 | n+n |
| 7934 | 7 | 18 | 32 | 57 | 0 | 1 | 0 | 0 | 0 | 1 | n+n |
| 7935 | 11 | 15 | 30 | 56 | 0 | 1 | 0 | 0 | 0 | 1 | n+n |
| 7937 | 10 | 19 | 29 | 58 | 0 | 0 | 0 | 0 | 0 | 0 | n+n |
| 7938 | 9 | 13 | 30 | 52 | 0 | 1 | 0 | 0 | 0 | 1 | n+n |
| 7939 | 9 | 15 | 29 | 53 | 0 | 1 | 0 | 0 | 0 | 1 | n+n |
| 7940 | 7 | 16 | 32 | 55 | 0 | 3 | 0 | 0 | 0 | 3 | n+n |
| 7941 | 8 | 16 | 31 | 55 | 0 | 0 | 0 | 0 | 0 | 0 | n+n |
| 7942 | 10 | 17 | 31 | 58 | 0 | 0 | 0 | 1 | 0 | 1 | n+n |
| 7943 | 8 | 18 | 31 | 57 | 0 | 0 | 1 | 0 | 0 | 1 | n+n |
| 7944 | 9 | 16 | 30 | 55 | 0 | 0 | 0 | 0 | 0 | 0 | n+n |
| 7945 | 11 | 17 | 29 | 57 | 2 | 1 | 0 | 0 | 0 | 3 | n+n |
| 7946 | 10 | 17 | 31 | 58 | 0 | 1 | 0 | 0 | 0 | 1 | n+n |
| 7947 | 9 | 16 | 30 | 55 | 0 | 0 | 0 | 0 | 0 | 0 | n+n |
| 7950 | 9 | 14 | 30 | 53 | 0 | 1 | 0 | 0 | 0 | 1 | n+n |
| 7951 | 20 | 33 | 39 | 92 | 0 | 0 | 0 | 0 | 0 | 0 | 2n+n |
| 7952 | 10 | 16 | 29 | 55 | 2 | 0 | 0 | 0 | 0 | 2 | n+n |
| 7953 | 9 | 17 | 31 | 57 | 1 | 3 | 0 | 0 | 0 | 4 | n+n |
| 7954 | 14 | 16 | 25 | 55 | 3 | 3 | 0 | 0 | 0 | 6 | n+n |
| 7955 | 8 | 16 | 31 | 55 | 0 | 0 | 0 | 0 | 0 | 0 | n+n |
| 7956 | 10 | 18 | 28 | 56 | 0 | 1 | 0 | 0 | 0 | 1 | n+n |
| 7957 | 9 | 15 | 30 | 54 | 0 | 0 | 0 | 0 | 0 | 0 | n+n |
| 7958 | 10 | 16 | 29 | 55 | 0 | 0 | 0 | 0 | 0 | 0 | n+n |
| 7959 | 12 | 16 | 27 | 55 | 2 | 1 | 0 | 0 | 0 | 3 | n+n |
| 7961 | 11 | 17 | 28 | 56 | 0 | 1 | 0 | 1 | 0 | 2 | n+n |
| 7962 | 10 | 16 | 30 | 56 | 1 | 4 | 0 | 0 | 0 | 5 | n+n |
| 7966 | 10 | 16 | 30 | 56 | 0 | 0 | 0 | 0 | 0 | 0 | n+n |
| 7968 | 12 | 17 | 28 | 57 | 1 | 0 | 0 | 0 | 0 | 1 | n+n |
| MTP58 | 12 | 17 | 29 | 58 | 1 | 1 | 0 | 0 | 0 | 2 | n+n |
| Yu6 | 12 | 17 | 27 | 56 | 3 | 0 | 0 | 0 | 0 | 3 | n+n |
| MTP94 | 20 | 34 | 40 | 94 | 0 | 0 | 0 | 0 | 0 | 0 | 2n+n |

**Table S2. Karyotype summary of all pop99 population**

| Material | Parantal chromosomes | | | Total chromosomes | Translocation chromosomes | | | | | Total Translocations | Reproduction |
| --- | --- | --- | --- | --- | --- | --- | --- | --- | --- | --- | --- |
|  | M | T | P |  | MP | PM | TP | MT | PT |  |  |
| 991 | 9 | 17 | 38 | 64 | 0 | 0 | 0 | 0 | 0 | 0 | n+n |
| 992 | 12 | 17 | 20 | 49 | 0 | 0 | 0 | 0 | 0 | 0 | n |
| 993 | 12 | 16 | 37 | 65 | 0 | 0 | 0 | 0 | 0 | 0 | n+n |
| 994 | 15 | 16 | 35 | 66 | 0 | 0 | 0 | 0 | 0 | 0 | n+n |
| 995 | 9 | 16 | 41 | 66 | 0 | 0 | 0 | 0 | 0 | 0 | n+n |
| 996 | 11 | 16 | 30 | 57 | 0 | 0 | 0 | 0 | 0 | 0 | n+n |
| 997 | 8 | 17 | 39 | 64 | 0 | 0 | 0 | 0 | 0 | 0 | n+n |
| 9910 | 10 | 17 | 40 | 67 | 0 | 0 | 0 | 0 | 0 | 0 | n+n |
| 9911 | 10 | 16 | 40 | 66 | 0 | 0 | 0 | 0 | 0 | 0 | n+n |
| 9912 | 13 | 14 | 35 | 62 | 0 | 0 | 0 | 0 | 0 | 0 | n+n |
| 9913 | 16 | 16 | 32 | 64 | 0 | 0 | 0 | 0 | 0 | 0 | n+n |
| 9914 | 9 | 18 | 40 | 67 | 0 | 0 | 0 | 0 | 0 | 0 | n+n |
| 9915 | 10 | 15 | 38 | 63 | 1 | 0 | 0 | 0 | 1 | 2 | n+n |
| 9916 | 12 | 17 | 36 | 65 | 0 | 0 | 0 | 0 | 1 | 1 | n+n |
| 9917 | 8 | 15 | 40 | 63 | 0 | 0 | 0 | 0 | 0 | 0 | n+n |
| 9918 | 7 | 17 | 40 | 64 | 0 | 0 | 0 | 0 | 0 | 0 | n+n |
| 9919 | 11 | 16 | 40 | 67 | 0 | 0 | 0 | 0 | 0 | 0 | n+n |
| 9920 | 10 | 16 | 40 | 66 | 1 | 0 | 0 | 1 | 0 | 2 | n+n |
| 9921 | 13 | 15 | 39 | 67 | 0 | 0 | 0 | 0 | 0 | 0 | n+n |
| 9922 | 8 | 16 | 63 | 87 | 0 | 0 | 0 | 0 | 0 | 0 | n+n+n |
| 9923 | 10 | 17 | 41 | 68 | 2 | 1 | 0 | 0 | 0 | 3 | n+n |
| 9924 | 9 | 17 | 41 | 67 | 2 | 0 | 0 | 0 | 0 | 2 | n+n |
| 9930 | 10 | 16 | 38 | 64 | 2 | 3 | 0 | 0 | 0 | 5 | n+n |
| 9935 | 18 | 14 | 32 | 64 | 1 | 0 | 1 | 0 | 0 | 2 | n+n |
| 9936 | 16 | 17 | 30 | 63 | 1 | 2 | 0 | 0 | 0 | 3 | n+n |
| 9938 | 15 | 15 | 35 | 65 | 2 | 4 | 0 | 0 | 0 | 6 | n+n |
| 9939 | 10 | 17 | 38 | 65 | 0 | 0 | 0 | 0 | 0 | 0 | n+n |
| 9940 | 15 | 17 | 34 | 66 | 0 | 2 | 0 | 0 | 0 | 2 | n+n |
| 9941 | 15 | 16 | 35 | 66 | 3 | 1 | 0 | 0 | 1 | 5 | n+n |
| 9943 | 17 | 17 | 31 | 65 | 2 | 2 | 1 | 0 | 0 | 5 | n+n |
| 9944 | 18 | 18 | 29 | 65 | 5 | 2 | 1 | 0 | 0 | 8 | n+n |
| 9947 | 19 | 15 | 30 | 64 | 3 | 0 | 0 | 0 | 1 | 4 | n+n |
| 9948 | 14 | 15 | 31 | 60 | 2 | 5 | 0 | 0 | 0 | 7 | n+n |
| 9949 | 15 | 17 | 31 | 63 | 0 | 2 | 0 | 0 | 0 | 2 | n+n |
| 9951 | 14 | 16 | 34 | 64 | 2 | 2 | 0 | 0 | 0 | 4 | n+n |
| 9952 | 10 | 16 | 38 | 64 | 2 | 1 | 0 | 0 | 0 | 3 | n+n |
| 9953 | 9 | 16 | 39 | 64 | 2 | 3 | 0 | 0 | 0 | 5 | n+n |
| 9956 | 18 | 14 | 33 | 65 | 4 | 3 | 0 | 0 | 0 | 7 | n+n |
| 9957 | 12 | 17 | 34 | 63 | 2 | 1 | 0 | 0 | 0 | 3 | n+n |
| 9958 | 10 | 17 | 39 | 66 | 0 | 1 | 0 | 1 | 1 | 3 | n+n |

**Table S3.** Phenotypic characteristics of MTP74 and MTP94 after allopolyploidization

| traits | V182 | TZ07 | 9475 | MTP74 | MTP94 |
| --- | --- | --- | --- | --- | --- |
| plant height (cm) | - | 254.33±16.86a | 142.00±20.30b | 265.67±4.04a | 261.00±10.15a |
| grass length (cm) | - | 243.00±36.43a | 179.33±18.01b | 285.67±3.51a | 284.67±4.51a |
| leaf length (cm) | - | 89.17±7.32a | 59.33±8.08b | 73.50±3.04b | 70.00±3.61b |
| leaf width (cm) | - | 3.00±0.35b | 3.07±0.06b | 5.23±0.25a | 4.70±0.26a |
| green leaf index (%) | - | 68.44±5.88a | 30.07±17.60b | 51.37±7.40ab | 64.32±6.20a |
| brix (%) | - | 7.87±0.76ab | 9.60±0.20a | 6.53±1.29b | 7.27±0.83b |
| branch number | - | 5.33±1.53b | 9.33±1.15a | 4.33±0.58b | 5.00±1.00b |
| number of nodes with aerial root | - | 0.00±0.00b | 6.67±0.58a | 5.67±3.79a | 2.67±0.58ab |
| node number | - | 10.33±2.08b | 17.00±1.00a | 16.67±3.06a | 14.00±1.00ab |
| internode (cm) | - | 18.67±4.07b | 7.33±1.53a | 16.03±1.55b | 15.23±1.50b |
| stem diameter (mm) | - | 11.29±1.31c | 13.90±1.92bc | 18.39±1.32a | 16.42±1.47ab |
| basal circumference (cm) | - | 73.00±6.08b | 143.00±18.25a | 85.00±6.24b | 82.33±8.02b |
| basal diameter (cm) | - | 23.67±5.77b | 47.83±5.35a | 27.67±5.51b | 28.33±4.73b |
| tiller number | - | 54.00±7.00a | 54.67±4.16a | 19.00±4.36b | 20.33±4.51b |
| fresh weight (kg·pl^-1^) | 0.70±0.04c | 1.17±0.34c | 4.80±0.79b | 6.78±0.61a | 6.92±0.41a |

Note: -: Not measured. Means marked with the same letter are not significantly different at the 0.05 probability level. Data are presented as mean ± SD. The same below.

**Table S4.** Phenotypic characteristics of the pop79 population

| traits | mean | SD | min | max | range | CV(%) |
| --- | --- | --- | --- | --- | --- | --- |
| plant height (cm) | 242.71 | 32.13 | 161.67 | 317.67 | 156.00 | 13.24 |
| grass length (cm) | 282.05 | 35.41 | 200.00 | 343.67 | 143.67 | 12.55 |
| leaf length (cm) | 81.76 | 11.54 | 58.33 | 109.33 | 51.00 | 14.11 |
| leaf width (cm) | 5.06 | 0.81 | 3.43 | 7.40 | 3.97 | 16.01 |
| green leaf index (%) | 36.07 | 16.51 | 0.00 | 69.49 | 69.49 | 45.77 |
| brix (%) | 8.46 | 1.18 | 5.93 | 12.13 | 6.20 | 13.95 |
| branch number | 5.19 | 1.52 | 2.67 | 10.67 | 8.00 | 29.29 |
| number of nodes with aerial root | 3.41 | 2.35 | 0.33 | 14.00 | 13.67 | 68.91 |
| node number | 15.67 | 2.59 | 11.00 | 23.67 | 12.67 | 16.53 |
| internode (cm) | 15.99 | 2.27 | 10.57 | 23.63 | 13.07 | 14.20 |
| stem diameter (mm) | 17.31 | 2.68 | 11.25 | 23.51 | 12.26 | 15.48 |
| basal circumference (cm) | 99.29 | 21.69 | 30.67 | 151.33 | 120.67 | 21.85 |
| basal diameter (cm) | 34.95 | 7.57 | 12.67 | 57.33 | 44.67 | 21.66 |
| tiller number | 29.43 | 10.01 | 6.00 | 62.00 | 56.00 | 34.01 |
| fresh weight (kg·pl^-1^) | 8.24 | 2.86 | 0.97 | 14.62 | 13.65 | 34.71 |

**Table S5.** Heterosis of phenotypic traits in pop79 population

| traits | MPH | | | | | BPH | | | | | |
| --- | --- | --- | --- | --- | --- | --- | --- | --- | --- | --- | --- |
|  | + | - | min | mean | max | + | - | min | mean | max |  |
| plant height (cm) | 57 | 8 | -20.69 | 19.07 | 55.85 | 15 | 50 | -39.15 | -8.64 | 19.57 |  |
| grass length (cm) | 59 | 6 | -13.98 | 21.31 | 47.81 | 31 | 34 | -29.99 | -1.26 | 20.30 |  |
| leaf length (cm) | 60 | 5 | -12.17 | 23.10 | 64.62 | 49 | 16 | -20.63 | 11.24 | 48.75 |  |
| leaf width (cm) | 56 | 9 | -17.27 | 21.98 | 78.31 | 27 | 37 | -34.39 | -3.27 | 41.40 |  |
| green leaf index (%) | 29 | 36 | -100.0 | -11.41 | 70.65 | 12 | 53 | -100.0 | -29.78 | 35.27 |  |
| brix (%) | 41 | 24 | -26.45 | 4.82 | 50.41 | 9 | 55 | -38.19 | -11.92 | 26.39 |  |
| branch number | 8 | 57 | -60.98 | -24.05 | 56.1 | 1 | 64 | -71.43 | -44.40 | 14.29 |  |
| number of nodes with aerial root | 6 | 59 | -94.59 | -44.78 | 127.03 | 6 | 59 | -95.00 | -48.92 | 110.00 |  |
| node number | 19 | 46 | -34.65 | -6.90 | 40.59 | 18 | 46 | -35.29 | -7.81 | 39.22 |  |
| internode (cm) | 62 | 3 | -9.56 | 36.87 | 102.28 | 30 | 34 | -34.10 | -0.27 | 47.40 |  |
| stem diameter (mm) | 45 | 20 | -30.34 | 7.24 | 45.62 | 21 | 44 | -38.83 | -5.83 | 27.86 |  |
| basal circumference (cm) | 15 | 50 | -73.10 | -12.90 | 32.75 | 2 | 63 | -78.55 | -30.56 | 5.83 |  |
| basal diameter (cm) | 18 | 47 | -66.45 | -7.40 | 51.88 | 3 | 62 | -73.52 | -26.92 | 19.86 |  |
| tiller number | 15 | 50 | -83.71 | -20.11 | 68.33 | 1 | 64 | -89.02 | -46.17 | 13.41 |  |
| fresh weight (kg·pl^-1^) | 52 | 13 | -83.31 | 42.24 | 152.37 | 47 | 18 | -85.75 | 21.44 | 115.48 |  |

Note：+: Number of positive combinations; -: Number of negative combinations. The same below.

**Table S6.** Phenotypic characteristics of the pop99 population

| traits | mean | SD | min | max | range | CV(%) |
| --- | --- | --- | --- | --- | --- | --- |
| plant height (cm) | 259.15 | 40.60 | 73.33 | 310.33 | 237.00 | 15.67 |
| grass length (cm) | 289.67 | 38.28 | 140.83 | 352.33 | 211.50 | 13.22 |
| leaf length (cm) | 76.47 | 9.99 | 53.33 | 106.67 | 53.33 | 13.06 |
| leaf width (cm) | 4.84 | 0.78 | 2.67 | 6.43 | 3.77 | 16.12 |
| green leaf index (%) | 33.59 | 15.97 | 2.22 | 74.53 | 72.31 | 47.54 |
| brix (%) | 8.20 | 1.41 | 4.33 | 11.4 | 7.07 | 17.20 |
| branch number | 5.23 | 1.47 | 0.33 | 9.67 | 9.33 | 28.11 |
| number of nodes with aerial root | 4.12 | 1.83 | 0.33 | 8.00 | 7.67 | 44.42 |
| node number | 16.64 | 2.12 | 11.67 | 21.33 | 9.67 | 12.74 |
| internode (cm) | 15.23 | 2.39 | 9.33 | 19.27 | 9.93 | 15.69 |
| stem diameter (mm) | 16.49 | 1.95 | 12.79 | 22.45 | 9.66 | 11.83 |
| basal circumference (cm) | 100.91 | 22.99 | 37.00 | 144.33 | 107.33 | 22.78 |
| basal diameter (cm) | 34.53 | 6.66 | 15.67 | 47.00 | 31.33 | 19.29 |
| tiller number | 28.52 | 11.84 | 9.00 | 61.00 | 52.00 | 41.51 |
| fresh weight (kg·pl^-1^) | 8.40 | 3.19 | 1.83 | 14.50 | 12.67 | 37.98 |

**Table S7.** Heterosis of phenotypic traits in pop99 population

| traits | MPH | | | | | | BPH | | | | |
| --- | --- | --- | --- | --- | --- | --- | --- | --- | --- | --- | --- |
|  | + | | - | min | mean | max | + | - | min | mean | max |
| plant height (cm) | | 51 | 3 | -63.61 | 28.61 | 54.01 | 30 | 24 | -71.90 | -0.71 | 18.90 |
| grass length (cm) | | 50 | 4 | -39.30 | 24.86 | 51.87 | 34 | 20 | -50.53 | 1.76 | 23.77 |
| leaf length (cm) | | 49 | 5 | -17.53 | 18.26 | 64.95 | 40 | 12 | -23.81 | 9.25 | 52.38 |
| leaf width (cm) | | 50 | 4 | -31.33 | 24.72 | 65.67 | 28 | 24 | -43.26 | 3.05 | 36.88 |
| green leaf index (%) | | 9 | 45 | -95.29 | -28.83 | 57.92 | 3 | 51 | -96.54 | -47.78 | 15.87 |
| brix (%) | | 23 | 31 | -48.62 | -2.75 | 35.18 | 8 | 44 | -54.86 | -14.57 | 18.75 |
| branch number | | 3 | 51 | -95.35 | -27.05 | 34.88 | 1 | 53 | -96.43 | -43.98 | 3.57 |
| number of nodes with aerial root | | 15 | 36 | -92.86 | -11.64 | 71.43 | 7 | 47 | -95.00 | -38.15 | 20.00 |
| node number | | 37 | 17 | -24.73 | 7.37 | 37.63 | 22 | 32 | -31.37 | -2.11 | 25.49 |
| internode (cm) | | 49 | 5 | -17.28 | 35.01 | 70.75 | 31 | 23 | -38.73 | 0.00 | 26.48 |
| stem diameter (mm) | | 39 | 15 | -15.64 | 8.76 | 48.05 | 27 | 26 | -22.11 | 0.43 | 36.70 |
| basal circumference (cm) | | 18 | 36 | -67.16 | -10.43 | 28.11 | 1 | 53 | -74.13 | -29.43 | 0.93 |
| basal diameter (cm) | | 14 | 40 | -58.86 | -9.32 | 23.41 | 0 | 54 | -67.25 | -27.80 | -1.74 |
| tiller number | | 9 | 45 | -76.00 | -23.95 | 62.67 | 3 | 51 | -83.54 | -47.83 | 11.59 |
| fresh weight (kg·pl^-1^) | | 43 | 11 | -68.71 | 43.49 | 156.39 | 36 | 18 | -73.49 | 21.54 | 117.16 |

**Table S8.** Comparison of traits between pop79 and pop99 populations

| traits | Population | | |
| --- | --- | --- | --- |
|  | pop79 | pop99 |  |
| plant height (cm) | 242.71±32.13b | 259.15±40.60a |  |
| grass length (cm) | 282.05±35.41a | 289.67±38.28a |  |
| leaf length (cm) | 81.76±11.54a | 76.47±9.99b |  |
| leaf width (cm) | 5.06±0.81a | 4.84±0.78a |  |
| green leaf index (%) | 36.07±16.51a | 33.59±15.97a |  |
| brix (%) | 8.46±1.18a | 8.20±1.41a |  |
| branch number | 5.19±1.52a | 5.23±1.47a |  |
| number of nodes with aerial root | 3.41±2.35a | 4.12±1.83a |  |
| node number | 15.67±2.59b | 16.64±2.12a |  |
| internode (cm) | 15.99±2.27a | 15.23±2.39a |  |
| stem diameter (mm) | 17.31±2.68a | 16.49±1.95a |  |
| basal circumference (cm) | 99.29±21.69a | 100.91±22.99a |  |
| basal diameter (cm) | 34.95±7.57a | 34.53±6.66a |  |
| tiller number | 29.43±10.01a | 28.52±11.84a |  |
| fresh weight (kg·pl^-1^) | 8.24±2.86a | 8.40±3.19a |  |

**Table S9.** Summary of the RNA-seq data

| Sample ID | Raw data  (Gb) | Clean data  (Gb) | Effective  Rate (%) | Q20  (%) | Q30  (%) | GC  (%) | Total mapped  (%) |
| --- | --- | --- | --- | --- | --- | --- | --- |
| 9475-1 | 12.55 | 12.49 | 99.52 | 98.12 | 94.46 | 56.39 | 87.60 |
| 9475-2 | 13.85 | 13.79 | 99.57 | 98.19 | 94.62 | 56.26 | 87.48 |
| 9475-3 | 11.72 | 11.67 | 99.57 | 98.26 | 94.83 | 55.97 | 86.94 |
| MTP74-1 | 14.13 | 14.05 | 99.43 | 97.93 | 94.03 | 54.78 | 87.20 |
| MTP74-2 | 10.49 | 10.43 | 99.43 | 97.78 | 93.72 | 53.56 | 84.51 |
| MTP74-3 | 11.47 | 11.40 | 99.39 | 97.90 | 93.91 | 54.06 | 83.93 |
| MTP94-1 | 15.37 | 15.19 | 98.83 | 97.89 | 94.17 | 56.54 | 87.66 |
| MTP94-2 | 13.92 | 13.77 | 98.92 | 97.92 | 94.21 | 54.91 | 86.85 |
| MTP94-3 | 16.17 | 15.95 | 98.64 | 97.82 | 93.97 | 55.84 | 87.07 |
| MTP56-1 | 14.32 | 14.24 | 99.44 | 98.01 | 94.19 | 55.97 | 88.38 |
| MTP56-2 | 13.50 | 13.40 | 99.26 | 97.61 | 93.25 | 54.82 | 83.50 |
| MTP56-3 | 13.54 | 13.43 | 99.19 | 97.60 | 93.29 | 55.93 | 84.94 |
| MTP67-1 | 11.58 | 11.51 | 99.40 | 97.61 | 93.21 | 56.62 | 88.24 |
| MTP67-2 | 13.88 | 13.79 | 99.35 | 97.77 | 93.66 | 56.39 | 87.30 |
| MTP67-3 | 12.62 | 12.54 | 99.37 | 97.43 | 92.83 | 56.79 | 87.18 |
| MTP57-1 | 12.40 | 12.33 | 99.44 | 97.17 | 92.09 | 54.39 | 85.21 |
| MTP57-2 | 12.10 | 12.03 | 99.42 | 97.12 | 91.98 | 55.26 | 85.40 |
| MTP57-3 | 11.53 | 11.48 | 99.57 | 97.09 | 91.83 | 54.26 | 84.72 |
| MTP66-1 | 11.08 | 10.98 | 99.10 | 97.49 | 93.15 | 57.00 | 88.81 |
| MTP66-2 | 11.15 | 11.05 | 99.10 | 97.68 | 93.45 | 56.60 | 88.24 |
| MTP66-3 | 14.58 | 14.47 | 99.25 | 97.53 | 93.25 | 56.92 | 88.39 |

**Table S10.** Identify of gene expression levels of different materials

| Samples | FPKM | | | | | |
| --- | --- | --- | --- | --- | --- | --- |
|  | 0~0.1 | 0.1~1 | 1~5 | 5~15 | 15~60 | >60 |
| 9475-1 | 43.84 | 25.96 | 19.39 | 6.74 | 3.10 | 0.97 |
| 9475-2 | 43.89 | 25.95 | 19.49 | 6.64 | 3.04 | 1.01 |
| 9475-3 | 45.63 | 26.07 | 18.09 | 6.16 | 3.00 | 1.05 |
| Mean | 44.45 | 25.99 | 18.99 | 6.51 | 3.05 | 1.01 |
| MTP74-1 | 26.10 | 42.38 | 21.96 | 5.90 | 2.72 | 0.94 |
| MTP74-2 | 27.69 | 40.94 | 21.65 | 6.01 | 2.78 | 0.93 |
| MTP74-3 | 29.05 | 39.92 | 21.18 | 5.92 | 2.94 | 0.98 |
| Mean | 27.61 | 41.08 | 21.60 | 5.94 | 2.81 | 0.95 |
| MTP94-1 | 33.03 | 40.44 | 18.19 | 5.00 | 2.40 | 0.94 |
| MTP94-2 | 27.67 | 40.47 | 22.00 | 6.09 | 2.79 | 0.97 |
| MTP94-3 | 27.74 | 41.78 | 21.09 | 5.72 | 2.66 | 1.00 |
| Mean | 29.48 | 40.90 | 20.43 | 5.60 | 2.62 | 0.97 |
| MTP56-1 | 27.23 | 35.85 | 25.60 | 7.26 | 3.17 | 0.89 |
| MTP56-2 | 36.92 | 32.75 | 19.81 | 6.38 | 3.12 | 1.02 |
| MTP56-3 | 36.60 | 34.65 | 19.22 | 5.81 | 2.71 | 1.00 |
| Mean | 33.59 | 34.42 | 21.54 | 6.48 | 3.00 | 0.97 |
| MTP67-1 | 30.60 | 38.26 | 22.01 | 5.75 | 2.51 | 0.87 |
| MTP67-2 | 31.94 | 37.98 | 20.88 | 5.71 | 2.57 | 0.91 |
| MTP67-3 | 32.14 | 38.08 | 20.68 | 5.65 | 2.54 | 0.91 |
| Mean | 31.56 | 38.11 | 21.19 | 5.70 | 2.54 | 0.90 |
| MTP57-1 | 32.74 | 31.91 | 25.47 | 6.95 | 2.27 | 0.66 |
| MTP57-2 | 33.17 | 31.70 | 25.24 | 7.00 | 2.26 | 0.63 |
| MTP57-3 | 32.41 | 30.91 | 26.20 | 7.45 | 2.39 | 0.63 |
| Mean | 32.78 | 31.51 | 25.64 | 7.13 | 2.30 | 0.64 |
| MTP66-1 | 30.01 | 38.57 | 22.46 | 5.73 | 2.40 | 0.82 |
| MTP66-2 | 29.53 | 37.96 | 23.17 | 5.99 | 2.51 | 0.83 |
| MTP66-3 | 31.04 | 37.76 | 22.27 | 5.70 | 2.40 | 0.84 |
| Mean | 30.19 | 38.10 | 22.63 | 5.81 | 2.44 | 0.83 |

**Table S11.** KEGG pathway of core DEGs in heterosis combination

MTP74 × 9475→MTP56

| KEGG pathway | Gene number | FDR |
| --- | --- | --- |
| Benzoxazinoid biosynthesis | 25 | 4.02369E-08 |
| Biosynthesis of amino acids | 148 | 2.8046E-06 |
| Protein processing in endoplasmic reticulum | 151 | 4.09828E-05 |
| Cysteine and methionine metabolism | 74 | 4.09828E-05 |
| Selenocompound metabolism | 25 | 0.0005 |
| Biosynthesis of various plant secondary metabolites | 43 | 0.0008 |
| beta-Alanine metabolism | 34 | 0.0048 |
| Protein export | 28 | 0.0075 |
| Taurine and hypotaurine metabolism | 10 | 0.0132 |
| Phagosome | 56 | 0.0161 |
| Biosynthesis of secondary metabolites | 540 | 0.0175 |
| Arginine and proline metabolism | 44 | 0.0180 |
| Peroxisome | 65 | 0.0235 |
| Photosynthesis - antenna proteins | 15 | 0.0235 |
| Sesquiterpenoid and triterpenoid biosynthesis | 10 | 0.0250 |
| Butanoate metabolism | 19 | 0.0290 |

**Table S12.** KEGG pathway of core DEGs in heterosis combination

MTP94 × 9475→MTP67

| KEGG pathway | Gene number | FDR |
| --- | --- | --- |
| Metabolic pathways | 1371 | 8.87E-05 |
| Benzoxazinoid biosynthesis | 25 | 8.87E-05 |
| Cysteine and methionine metabolism | 101 | 0.0001 |
| Tropane, piperidine and pyridine alkaloid biosynthesis | 35 | 0.0001 |
| Biosynthesis of various plant secondary metabolites | 59 | 0.0010 |
| Arginine and proline metabolism | 68 | 0.0018 |
| Flavonoid biosynthesis | 52 | 0.0018 |
| Glutathione metabolism | 56 | 0.0023 |
| Cyanoamino acid metabolism | 51 | 0.0045 |
| Biosynthesis of secondary metabolites | 824 | 0.0045 |
| Selenocompound metabolism | 29 | 0.0063 |
| Biosynthesis of amino acids | 184 | 0.0065 |
| alpha-Linolenic acid metabolism | 70 | 0.0346 |
| Pentose phosphate pathway | 55 | 0.0346 |
| Nitrogen metabolism | 26 | 0.0399 |

**Table S13.** KEGG pathway of core DEGs shared by two heterosis combination

| KEGG pathway | Gene number | FDR |
| --- | --- | --- |
| Protein processing in endoplasmic reticulum | 66 | 0.0001 |
| alpha-Linolenic acid metabolism | 30 | 0.0001 |
| Arginine and proline metabolism | 26 | 0.0003 |
| Cysteine and methionine metabolism | 33 | 0.0004 |
| Biosynthesis of various plant secondary metabolites | 20 | 0.0030 |
| Ubiquitin mediated proteolysis | 33 | 0.0030 |
| beta-Alanine metabolism | 17 | 0.0037 |
| Protein export | 13 | 0.0233 |
| Butanoate metabolism | 10 | 0.0279 |
| Photosynthesis - antenna proteins | 8 | 0.0279 |
| Taurine and hypotaurine metabolism | 5 | 0.0482 |

**Table S14.** KEGG pathway of core DEGs in weakness combination

MTP74 × 9475→MTP57

| KEGG pathway | Gene number | FDR |
| --- | --- | --- |
| Nitrogen metabolism | 54 | 4.90E-07 |
| Glutathione metabolism | 96 | 4.90E-07 |
| Porphyrin metabolism | 65 | 4.90E-07 |
| alpha-Linolenic acid metabolism | 130 | 4.90E-07 |
| Circadian rhythm - plant | 127 | 1.09E-05 |
| Stilbenoid, diarylheptanoid and gingerol biosynthesis | 32 | 4.10E-05 |
| Aflatoxin biosynthesis | 26 | 5.78E-05 |
| Benzoxazinoid biosynthesis | 32 | 5.78E-05 |
| Protein processing in endoplasmic reticulum | 307 | 0.0008 |
| Propanoate metabolism | 52 | 0.0013 |
| Pantothenate and CoA biosynthesis | 58 | 0.0025 |
| beta-Alanine metabolism | 66 | 0.0039 |
| Carotenoid biosynthesis | 35 | 0.0060 |
| Thiamine metabolism | 28 | 0.0146 |
| Pyruvate metabolism | 153 | 0.0188 |
| Photosynthesis - antenna proteins | 28 | 0.0200 |
| Ribosome | 110 | 0.0211 |
| Carbon metabolism | 329 | 0.0354 |

**Table S15.** KEGG pathway of core DEGs in weakness combination

MTP94 × 9475→MTP66

| KEGG pathway | Gene number | FDR |
| --- | --- | --- |
| Carbon fixation in photosynthetic organisms | 220 | 1.59E-23 |
| Photosynthesis | 90 | 1.22E-18 |
| Carbon metabolism | 390 | 2.51E-14 |
| Oxidative phosphorylation | 183 | 1.46E-13 |
| Glyoxylate and dicarboxylate metabolism | 147 | 3.17E-11 |
| Metabolic pathways | 2000 | 1.20E-09 |
| alpha-Linolenic acid metabolism | 123 | 1.06E-07 |
| Pyruvate metabolism | 168 | 9.84E-07 |
| Propanoate metabolism | 56 | 5.68E-06 |
| Biosynthesis of secondary metabolites | 1195 | 8.62E-05 |
| Aflatoxin biosynthesis | 24 | 8.62E-05 |
| Ribosome | 114 | 0.0002 |
| Linoleic acid metabolism | 120 | 0.0006 |
| Selenocompound metabolism | 39 | 0.0023 |
| Arginine and proline metabolism | 88 | 0.0032 |
| Tropane, piperidine and pyridine alkaloid biosynthesis | 38 | 0.0058 |
| Stilbenoid, diarylheptanoid and gingerol biosynthesis | 24 | 0.0068 |
| beta-Alanine metabolism | 59 | 0.0093 |
| Glycolysis / Gluconeogenesis | 165 | 0.0101 |
| Taurine and hypotaurine metabolism | 15 | 0.0182 |

**Table S16.** KEGG pathway of core DEGs shared by two weakness combinations

| KEGG pathway | Gene number | FDR |
| --- | --- | --- |
| alpha-Linolenic acid metabolism | 49 | 1.11E-06 |
| Pyruvate metabolism | 63 | 2.77E-06 |
| Propanoate metabolism | 26 | 2.77E-06 |
| Aflatoxin biosynthesis | 14 | 5.33E-06 |
| Carbon fixation in photosynthetic organisms | 56 | 6.70E-05 |
| Linoleic acid metabolism | 44 | 0.0009 |
| Photosynthesis | 22 | 0.0010 |
| Protein processing in endoplasmic reticulum | 92 | 0.0017 |
| Benzoxazinoid biosynthesis | 12 | 0.0022 |
| Metabolic pathways | 546 | 0.0066 |
| Carbon metabolism | 99 | 0.0085 |
| Biosynthesis of secondary metabolites | 340 | 0.0088 |
| Oxidative phosphorylation | 43 | 0.0335 |
| Glutathione metabolism | 24 | 0.0375 |

**Table S17.** Hub genes in four modules significantly associated with fresh-weight

| Module | Gene ID | Nr description | Weight | KME |
| --- | --- | --- | --- | --- |
| Salmon | i1_LQ_MTP1_c111783/f1p5/1318 | Tonoplast intrinsic protein 4-1 ZmTIP4;1 [Zea mays] | 0.32 | 0.93 |
| Salmon | i1_LQ_MTP1_c23286/f1p1/1487 | LRR receptor-like serine/threonine-protein kinase FLS2 [Zea mays] | 0.35 | 0.94 |
| Salmon | i1_LQ_MTP1_c24023/f1p1/1581 | 1 2-methoxy-6-polyprenyl-1,4-benzoquinol methylase [Zea mays] | 0.34 | 0.98 |
| Salmon | i3_LQ_MTP1_c14573/f1p0/3877 | SAC3 family protein A isoform X4 [Zea mays] | 0.33 | 0.97 |
| Salmon | i1_LQ_MTP1_c13349/f1p10/1399 | 60S ribosomal protein L3-1 [Zea mays] | 0.34 | 0.95 |
| Salmon | i2_HQ_MTP1_c39046/f3p3/2900 | Prolyl oligopeptidase family protein, partial [Zea mays] | 0.31 | 0.98 |
| Salmon | i4_LQ_MTP1_c9185/f1p0/4486 | Prolyl oligopeptidase family protein, partial [Zea mays] | 0.31 | 0.98 |
| Salmon | i2_LQ_MTP1_c28372/f1p4/2891 | phosphoinositide phosphatase SAC2 isoform X1 [Zea mays] | 0.32 | 0.96 |
| Salmon | i1_LQ_MTP1_c14799/f1p7/1900 | putative TCP-1/cpn60 chaperonin family protein [Zea mays] | 0.30 | 0.98 |
| Salmon | i2_LQ_MTP1_c88227/f1p7/2075 | HGA4 isoform X1 [Zea mays] | 0.31 | 0.98 |
| Greenyellow | i3_HQ_MTP1_c14175/f4p26/3329 | unnamed protein product [Miscanthus lutarioriparius] | 0.37 | 0.94 |
| Greenyellow | i2_LQ_MTP1_c25337/f1p0/2410 | heat shock 70 kDa protein [Zea mays] | 0.39 | 0.98 |
| Greenyellow | i2_HQ_MTP1_c104163/f13p0/2396 | probable mediator of RNA polymerase II transcription subunit 37c [Sorghum bicolor] | 0.39 | 0.95 |
| Greenyellow | i2_LQ_MTP1_c40910/f1p11/2364 | probable mediator of RNA polymerase II transcription subunit 37c [Sorghum bicolor] | 0.38 | 0.97 |
| Greenyellow | i2_LQ_MTP1_c54387/f1p0/2377 | heat shock 70 kDa protein [Zea mays] | 0.38 | 0.98 |
| Brown | i1_LQ_MTP1_c102947/f2p9/1919 | uncharacterized protein LOC100275604 [Zea mays] | 0.49 | 0.97 |
| Brown | i1_LQ_MTP1_c26384/f1p11/1567 | Polyubiquitin-A [Tetrabaena socialis] | 0.48 | 0.99 |
| Brown | i1_LQ_MTP1_c40944/f1p0/1484 | hypothetical protein BDA96_10G310200 [Sorghum bicolor] | 0.48 | 0.98 |
| Brown | i1_HQ_MTP1_c120675/f17p4/1612 | polyubiquitin [Sorghum bicolor] | 0.48 | 0.98 |
| Brown | i1_LQ_MTP1_c70451/f1p12/1633 | unnamed protein product [Miscanthus lutarioriparius] | 0.48 | 0.97 |
| Blue | i2_HQ_MTP1_c135335/f7p0/2055 | ABC transporter F family member 1 [Zea mays] | 0.49 | 0.99 |
| Blue | i3_LQ_MTP1_c16521/f1p0/3047 | zeaxanthin epoxidase, chloroplastic isoform X1 [Zea mays] | 0.49 | 0.99 |
| Blue | i2_HQ_MTP1_c81462/f2p0/2148 | hypothetical protein SORBI_3006G135600 [Sorghum bicolor] | 0.48 | 0.99 |
| Blue | i3_LQ_MTP1_c11860/f1p0/3820 | Vacuolar protein sorting-associated protein 41 homolog [Zea mays] | 0.48 | 0.99 |
| Blue | i2_LQ_MTP1_c72576/f1p0/2208 | unknown [Zea mays] | 0.48 | 0.99 |

**Table S18.** Fresh yield of perennial forage maize variety in different ecological zones (t·ha^-1^)

| Locations | Materials | Cutting frequency | 2019 | 2020 | 2021 | 2022 |
| --- | --- | --- | --- | --- | --- | --- |
| Chengdu | Silage maize | Total | 52.13±0.73 | 52.74±1.27 | 54.43±1.78 | 54.37±1.56 |
|  | Yucao 6 | 1^st^ | 90.78±1.27 | 94.98±1.73 | 94.58±0.91 | 89.76±1.53 |
|  |  | 2^st^ | 42.43±1.35 | 45.85±1.71 | 41.37±2.11 | 38.95±1.44 |
|  |  | Total | 133.21 | 140.83 | 135.95 | 128.71 |
|  | Yucao 9919 | 1^st^ | 99.39±2.10 | 104.28±2.23 | 99.33±1.88 | 97.11±1.52 |
|  |  | 2^st^ | 50.19±1.32 | 51.18±1.25 | 47.13±1.46 | 45.91±1.53 |
|  |  | Total | 149.58 | 155.46 | 146.47 | 143.03 |
| Nanchong | Silage maize | Total | 55.95±1.31 | 52.96±1.35 | 57.93±1.91 | 51.93±1.72 |
|  | Yucao 6 | 1^st^ | 93.04±1.12 | 95.70±1.73 | 91.50±1.97 | 86.93±1.47 |
|  |  | 2^st^ | 46.25±1.05 | 44.27±1.74 | 43.22±0.80 | 39.93±2.10 |
|  |  | Total | 139.29 | 139.97 | 134.72 | 126.86 |
|  | Yucao 9919 | 1^st^ | 104.12±1.80 | 108.16±2.36 | 97.91±1.28 | 90.58±1.96 |
|  |  | 2^st^ | 47.17±1.66 | 49.98±2.09 | 41.98±0.85 | 40.36±1.33 |
|  |  | Total | 151.28 | 158.14 | 139.89 | 130.94 |
| Liangshan | Silage maize | Total | 40.23±1.20 | 42.21±1.19 | 39.05±1.43 | 44.07±1.17 |
|  | Yucao 6 | 1^st^ | 74.97±2.02 | 79.79±1.35 | 80.21±2.03 | 72.49±1.10 |
|  |  | 2^st^ | 37.37±1.62 | 38.10±1.80 | 35.92±2.29 | 33.75±0.97 |
|  |  | Total | 112.34 | 117.89 | 116.13 | 106.24 |
|  | Yucao 9919 | 1^st^ | 90.94±1.86 | 95.15±1.21 | 94.55±1.97 | 89.26±1.14 |
|  |  | 2^st^ | 42.73±1.55 | 44.95±1.65 | 41.13±0.84 | 37.80±0.98 |
|  |  | Total | 133.66 | 140.10 | 135.68 | 127.06 |

**Table S19.** Hay yield of perennial forage maize variety in different ecological zones (t·ha^-1^)

| Locations | Materials | Cutting frequency | 2019 | 2020 | 2021 | 2022 |
| --- | --- | --- | --- | --- | --- | --- |
| Chengdu | Silage maize | Total | 15.77±0.22 | 15.51±0.37 | 17.09±0.56 | 17.49±0.50 |
|  | Yucao 6 | 1^st^ | 18.62±0.26 | 17.95±0.33 | 18.54±0.18 | 16.34±0.28 |
|  |  | 2^st^ | 7.21±0.23 | 8.21±0.31 | 8.85±0.45 | 7.56±0.28 |
|  |  | Total | 25.83 | 26.16 | 27.39 | 23.89 |
|  | Yucao 9919 | 1^st^ | 19.76±0.42 | 20.23±0.43 | 18.58±0.35 | 18.65±0.29 |
|  |  | 2^st^ | 9.29±0.24 | 9.72±0.24 | 8.39±0.26 | 8.54±0.28 |
|  |  | Total | 29.04 | 29.96 | 26.97 | 143.03 |
| Nanchong | Silage maize | Total | 17.34±0.35 | 16.10±0.41 | 16.92±0.56 | 16.26±0.54 |
|  | Yucao 6 | 1^st^ | 18.14±0.22 | 18.47±0.33 | 17.20±0.37 | 16.60±0.28 |
|  |  | 2^st^ | 8.74±0.20 | 8.01±0.32 | 8.21±0.15 | 7.75±0.41 |
|  |  | Total | 26.88 | 26.48 | 25.41 | 27.19 |
|  | Yucao 9919 | 1^st^ | 18.64±0.32 | 19.90±0.43 | 18.60±0.24 | 16.12±0.35 |
|  |  | 2^st^ | 9.06±0.32 | 8.95±0.37 | 7.81±0.16 | 7.31±0.24 |
|  |  | Total | 27.69 | 28.85 | 26.41 | 23.43 |
| Liangshan | Silage maize | Total | 13.76±0.41 | 13.30±0.37 | 12.89±0.47 | 13.70±0.36 |
|  | Yucao 6 | 1^st^ | 13.04±0.35 | 14.36±0.24 | 15.08±0.38 | 13.48±0.20 |
|  |  | 2^st^ | 6.73±0.29 | 6.74±0.32 | 6.90±0.44 | 6.18±0.18 |
|  |  | Total | 19.77 | 21.11 | 21.98 | 19.66 |
|  | Yucao 9919 | 1^st^ | 16.00±0.33 | 17.22±0.22 | 17.49±0.36 | 17.32±0.22 |
|  |  | 2^st^ | 7.82±0.28 | 8.45±0.31 | 7.82±0.16 | 6.88±0.18 |
|  |  | Total | 23.82 | 25.67 | 35.31 | 24.20 |

**Table S20.** Mean hay and fresh yield of perennial forage maize variety in different ecological zones (t·ha^-1^)

| Locations | Materials | 2019 | 2020 | 2021 | 2022 | Mean yield (FW/DW)  (t·ha^-1^) |
| --- | --- | --- | --- | --- | --- | --- |
| Chengdu | Silage maize | 52.13/15.77 | 52.74/15.50 | 54.43/17.09 | 54.15/17.49 | 53.37/16.46 |
|  | Yucao 6 | 133.21/25.83 | 140.83/26.16 | 135.95/27.39 | 128.71/23.89 | 134.67/25.82 |
|  | Yucao 9919 | 149.58/29.04 | 155.46/29.96 | 146.47/26.96 | 143.03/27.19 | 148.64/28.29 |
| Nanchong | Silage maize | 55.95/17.34 | 52.96/16.10 | 57.93/16.92 | 51.93/16.26 | 54.69/16.65 |
|  | Yucao 6 | 139.29/26.88 | 139.97/26.48 | 134.72/25.41 | 126.86/24.35 | 135.21/25.78 |
|  | Yucao 9919 | 151.28/27.69 | 158.14/28.85 | 139.89/26.41 | 130.94/23.43 | 145.06/26.60 |
| Nanchong | Silage maize | 40.23/13.76 | 42.21/13.30 | 39.05/12.89 | 44.07/13.70 | 41.39/13.41 |
|  | Yucao 6 | 112.34/19.77 | 117.89/21.11 | 116.13/21.98 | 106.24/19.66 | 113.15/20.63 |
|  | Yucao 9919 | 133.66/23.82 | 140.10/25.67 | 135.68/25.31 | 127.06/24.20 | 134.13/24.75 |
| number of replication | Silage maize | 3 | 3 | 3 | 3 | 12 |
|  | Yucao 6 | 3 | 3 | 3 | 3 | 12 |
|  | Yucao 9919 | 3 | 3 | 3 | 3 | 12 |
| Mean yield (FW/DW)  (t·ha^-1^) | Silage maize | 49.44/15.62 | 49.30/14.97 | 50.47/15.63 | 50.05/15.82 | 49.82/15.51 |
|  | Yucao 6 | 128.28/24.16 | 132.90/24.58 | 128.93/24.93 | 121.32/22.77 | 127.68/24.08 |
|  | Yucao 9919 | 144.84/26.85 | 151.24/28.16 | 140.68/26.23 | 133.68/24.94 | 142.61/26.54 |

**Figure S1.** MPH for each trait in pop79 population and pop99 population, respectively.

**Figure S2.** Analysis of panicle traits heterosis of representative materials

from two populations.

(a) - (f) represents the 6 panicle traits observed in the MTP74 × 9475 cross combination. (g) - (l) represents the 6 reproductive traits observed in the MTP94 × 9475 cross combination, and MPV represents the average of the parents of the trait. *n* = 10.

**Figure S3.** Analysis of cell and stomatal size heterosis in representative materials

of two populations.

(a) - (f) denote the six cell size-related traits observed in the MTP74 × 9475 hybrid combination. (i) - (p) denote the six cell size-related traits observed in the MTP94 × 9475 hybrid combination. MPV denotes the mean of the two parents for the trait. *n* ≥ 10.

**Figure S4.** Analysis of photosynthetic parameters heterosis of representative

materials of two populations.

(a) - (f) denote the six photosynthesis and chlorophyll content related metrics observed in the MTP74 × 9475 hybrid combination. (i) - (p) denote the six photosynthesis and chlorophyll content related metrics observed in the MTP94 × 9475 hybrid combination, and MPV denotes the mean of the two parents for the trait. *n* ≥ 3.

**Figure S5.** Pearson correlation analysis between important traits.

The numbers in the boxes represent correlation coefficients, and *, **, *** indicate *P* < 0.05, *P* < 0.01, and *P* < 0.001, respectively.

**Figure S6.** Effects of *Z. perennis* genome dosage on panicle traits of representative materials.

**Figure S7.** Effects of *Z. perennis* genome dosage on cell and stomatal size

of representative materials.

**Figure S8.** Effects of *Z. perennis* genome dosage on photosynthesis of representative materials.

**Figure S9.** Identify of gene expression levels of different materials.

**Figure S10.** Heatmap of correlation of gene expression between 21 samples.

**Figure S11.** DEGs triggered by hybridity and their functional annotation

in allopolyploids.

(a) Venn diagram representing the set of genes shared by the heterosis and hybrid weakness gene clusters; (b) GO function annotations for 324 DEGs triggered by hybridity; (c) GO function annotations for 1298 DEGs triggered by heterosis only; (d) GO function annotations for 2616 DEGs triggered by hybrid weakness only. The top 15 GO terms are illustrated in figure.

**Figure S12.** DEGs triggered by hybridity and their KEGG pathway in allopolyploids.

(a) Venn diagram representing the set of genes shared by the heterosis and hybrid weakness gene clusters; (b) KEGG pathway enrichment of 324 DEGs triggered by hybridity; (c) KEGG pathway enrichment for 1298 DEGs triggered by heterosis only; (d) KEGG pathway enrichment for 2616 DEGs triggered by hybrid weakness only. The top 15 GO terms are illustrated in figure.

**Figure S13.** Analysis of expression pattern and KEGG pathway of polyploid hybrid F_1_s in perennial forage maize.

(a) The number and proportion of genes present different expression patterns in the five polyploid hybrid F1s, with M and P denoting the maternal and the paternal of polyploid hybrids, respectively. (b)-(j) indicate the KEGG pathway enrichment results for the six non-additive expression pattern gene sets, and the top 10 pathways for that expression pattern were illustrated for each perennial forage maize line.

**Figure S14.** GO annotation of non-additively expressed gene sets from heterosis and hybrid weakness materials.

(a) and (d) indicate maternal expression level dominance genes. (b) and (e) represent paternal expression level dominance genes. (c) and (f) indicate transgressive upregulation expression genes and transgressive downregulation expression genes, respectively. The top 10 GO annotation terms for this expression pattern for each material are shown.

**Figure S15.** KEGG enrichment in four modules significantly correlated with FW.

**Figure S16.** Heatmap comparison of the DEGs for heterosis F_1_s (MTP56 and MTP67) and their female parents in protein quality control system, glutathione metabolism, spliceosome, energy and glucose metabolism, and metabolic pathway.

**Figure S17.** Co-expression network of blue and brown module genes

**Figure S18.** Validation of gene expression in negative correlation module at population level.

**Figure S19.** Expression of 205 dosage effect genes in representative materials of two populations

**Figure S20.** Detection of pollen fertility of the pop79 sing I₂‑KI staining.

**Figure S21.** Detection of pollen fertility of the pop99 sing I₂‑KI staining.

**Figure S22.** Comparison of pollen fertility between pop79 and pop99, error bars represent SEM.

**Figure S23.** Field scenes of the perennial forage maize.

(a) Allopolyploid trihybrids MTP74, MTP94 and their descendants MTP56, MTP67. (b)-(d) Through the systematic evaluation of elite trihybrids (MTP56 and MTP67, derived from pop79 and pop99) across diverse ecoregions, we successfully developed and commercialized two groundbreaking perennial forage maize cultivars Yucao 6 and Yucao 9919.
